# Multi-Omics Investigation into Acute Myocardial Infarction: An Integrative Method Revealing Interconnections amongst the Metabolome, Lipidome, Glycome, and Metallome

**DOI:** 10.3390/metabo12111080

**Published:** 2022-11-08

**Authors:** Si Ying Lim, Felicia Li Shea Lim, Inmaculada Criado-Navarro, Xin Hao Yeo, Hiranya Dayal, Sri Dhruti Vemulapalli, Song Jie Seah, Anna Karen Carrasco Laserna, Xiaoxun Yang, Sock Hwee Tan, Mark Y. Chan, Sam Fong Yau Li

**Affiliations:** 1 NUS Graduate School’s Integrative Sciences & Engineering Programme (ISEP), National University of Singapore, Singapore 119077, Singapore; 2 Department of Chemistry, National University of Singapore, Singapore 117543, Singapore; 3 Central Instrumentation Facility (Laguna Campus), Office of the Vice President for Research and Innovation, De La Salle University, Manila 1004, Philippines; 4 Cardiovascular Research Institute, Yong Loo Lin School of Medicine, National University of Singapore, Singapore 117599, Singapore

**Keywords:** multi-omics, metabolomics, lipidomics, glycomics, metallomics, acute myocardial infarction

## Abstract

Acute myocardial infarction (AMI) is a leading cause of mortality and morbidity worldwide. This work aims to investigate the translational potential of a multi-omics study (comprising metabolomics, lipidomics, glycomics, and metallomics) in revealing biomechanistic insights into AMI. Following the N-glycomics and metallomics studies performed by our group previously, untargeted metabolomic and lipidomic profiles were generated and analysed in this work via the use of a simultaneous metabolite/lipid extraction and liquid chromatography–tandem mass spectrometry (LC–MS/MS) analysis workflow. The workflow was applied to blood plasma samples from AMI cases (*n* = 101) and age-matched healthy controls (*n* = 66). The annotated metabolomic (number of features, *n* = 27) and lipidomic (*n* = 48) profiles, along with the glycomic (*n* = 37) and metallomic (*n* = 30) profiles of the same set of AMI and healthy samples were integrated and analysed. The integration method used here works by identifying a linear combination of maximally correlated features across the four omics datasets, via utilising both block-partial least squares-discriminant analysis (block-PLS-DA) based on sparse generalised canonical correlation analysis. Based on the multi-omics mapping of biomolecular interconnections, several postulations were derived. These include the potential roles of glycerophospholipids in N-glycan-modulated immunoregulatory effects, as well as the augmentation of the importance of Ca–ATPases in cardiovascular conditions, while also suggesting contributions of phosphatidylethanolamine in their functions. Moreover, it was shown that combining the four omics datasets synergistically enhanced the classifier performance in discriminating between AMI and healthy subjects. Fresh and intriguing insights into AMI, otherwise undetected via single-omics analysis, were revealed in this multi-omics study. Taken together, we provide evidence that a multi-omics strategy may synergistically reinforce and enhance our understanding of diseases.

## 1. Introduction

Acute myocardial infarction (AMI), a condition classified under coronary heart disease (CHD), is one of the leading causes of mortality and morbidity worldwide [1,2,3]. Novel and reliable biomarkers are needed to assist in risk assessment, accurate diagnosis and prognosis, and may also act as mediators of disease. Thus, more in-depth research, especially using ‘omics’ tools, is needed to better understand the biology of these novel biomarkers. For example, as shown by a recent bibliometric study, metabolomics and lipidomics have already been widely applied to study CHD, utilising targeted and/or untargeted methods [4].

Meanwhile, to build upon the large volumes of “single-omics” datasets that are already widely available, recent research trends highlight that there is a shift from a reductionist to a global approach regarding the application of omics approaches [4]. The exciting emergence of the data science field and the development of bioinformatics tools have made it possible to integrate data across various omics levels. When considering the four omics strategies selected here, metabolomics and lipidomics can be considered the “glue” between them, as their alterations may reflect modulations by both glycomics and metallomics as a downstream effect of impaired protein synthesis, enzyme/substrate activities, etc. Previous reports on metabolomics/lipidomics–glycomics and metabolomics/lipidomics–metallomics data, albeit very few in number, have shown promise [5,6,7]. The first benefit that can be reaped is the improved performance of discriminant models for disease predictions [8,9]. Pathway-centric and network-based approaches could also return the second benefit of revealing inherent chemical connections between the different biomolecules [5,6,7].

There is an opportunity and demand for integrative analysis of multi-omics data for heart disease research. In our previous works, comprehensive glycomic [10] and metallomic profiles [11] had been generated. In the current work, metabolomics and lipidomics datasets are supplemented through untargeted studies that will allow the exploration of multi-omics connections without restrictions. As a multi-omics study, any attempt to minimise sample consumption and optimise an efficient analytical workflow has merit. A lateral objective of this work is thus to capitalise on existing simultaneous extraction methods, with an application at a higher, multi-omics level. A Matyash-based method was utilised [12], as it has been previously shown to be optimal and suitable for untargeted extraction of lipids and metabolites in human blood plasma/serum [13,14].

Overall, this work aimed to enhance the process of biomarker discovery and provide insights into the biological pathways/networks of AMI via a multi-omics approach [15,16,17,18,19]. Such an ability to synergistically reprocess single-omics datasets in an integrative manner is enabled by using the same set of patient and control samples [9]. Since a multi-omics integration involving the combination of metabolomics, lipidomics, glycomics, and metallomics has yet to be endeavoured, this study can be considered a fresh and novel attempt to decipher the interconnected disturbances among the downstream omics levels which are pertinent to AMI.

## 2. Materials and Methods

### 2.1. Biospecimens from Clinical Studies

This study was approved by the National Healthcare Group Doman Specific Review Board (NHG DSRB; REF NO. 2013-00248 and 2016-00210), and all subjects gave their informed consent to participate prior to the inclusion. All experiments were performed in compliance with the relevant laws and institutional guidelines. Blood plasma samples from 101 AMI patients were collected from the National University Heart Centre, Tan Tock Seng Hospital, Changi General Hospital, Sarawak General Hospital Heart Centre, and Christchurch District Hospital. AMI plasma samples were collected 24–48 h post-percutaneous coronary intervention (PCI). Blood plasma samples from 66 matched healthy community-dwelling controls were collected from Singapore.

The details for sample collection and processing are as follows. From each subject, 18 mL of blood was drawn into labelled 3 × 6 mL K_2_-EDTA tubes. The blood in the tubes was immediately mixed by inversion 8–10 times. Blood collected in K_2_-EDTA tubes was then immediately centrifuged for 10 min at 4000 rpm at 4 °C to collect the plasma portion. Plasma samples collected from all three K_2_-EDTA tubes were pooled before aliquoting into 2 mL storage tubes for different experiments. All samples were stored at −80 °C prior to the various omics extraction experiments.

### 2.2. Untargeted Metabolomics and Lipidomics Analysis and Data Acquisition

#### 2.2.1. Reagents and Chemicals

The following comprises chemical/reagent information for the metabolomics and lipidomics analysis. As for the glycomics and metallomics analysis, details regarding the materials used can be found in their respective publications [10,11]. All reagents used were of analytical grade unless otherwise stated. Ultrapure water (18.2 Ω) used for all chemical and sample preparation was obtained from an Ultra Clear^TM^ water purification system by Siemenssie (Munich, Germany). For the mobile phases and extraction solvents used in the liquid chromatography–mass spectrometry (LC–MS)-based metabolomics/lipidomics analysis, LC–MS grade isopropanol, acetonitrile, methanol and formic acid were purchased from Fisher Chemicals (Waltham, MA, USA), and HPLC Plus grade tert-butyl methyl ether (MTBE) was purchased from Sigma Aldrich (St. Louis, MO, USA). For the internal standards used, SPLASH II Lipidomix Mass Spec Standard was purchased from Avanti Polar Lipids (Birmingham, AL, USA), and BOC–Leucine (99% for HPLC) standard was purchased from Sigma Aldrich.

#### 2.2.2. Sample Extraction and Preparation

The polar metabolite and non-polar lipid extracts used for the untargeted analysis were obtained simultaneously using the Matyash extraction method [12]. Prior to the extraction, 40 uL of blood plasma sample was thawed on ice. This was followed by the addition of extraction solvents: 300 μL of methanol containing SPLASH II Lipidomix as well as BOC–Leucine as internal standards, and 1 mL of MTBE. Samples were then incubated at room temperature in a Vortemp Shaking Incubator from UniEquip (Munich, Germany) (Munchen, DE) for 1 h. Next, 250 μL MS grade water was then added to induce phase separation, before centrifugation at 1000× *g* for 10 min. After phase separation, the upper organic phase was transferred to a new tube while the bottom layer was re-extracted with extraction solvents. After centrifugation, the two phases were carefully separated and dried at 4 °C in a SpeedVac Concentrator until the solvents were fully evaporated. A process blank was also prepared with the same preparation steps, with the use of ultrapure water in place of blood plasma.

Before the sample run, dried non-polar extracts used for lipidomics analysis were reconstituted in 300 μL of isopropanol, while dried polar extracts used for the metabolomics analysis were reconstituted in 50 μL 50% acetonitrile. Quality control (QC) samples were prepared by mixing 10 μL of each plasma sample and aliquots of the pooled QC sample were subjected to the same sample preparation procedure. This pooled QC sample also provided a representation of all the analytes present in the samples.

#### 2.2.3. LC-QTOF Analysis

For both the polar metabolite and non-polar lipid fractions, the samples were analysed on an Agilent 6540 UHD Accurate-Mass Q-TOF LC/MS coupled with an Agilent 1290 Infinity LC system and operated using the B.08.00 Agilent Mass Hunter Software from Agilent Technologies.

For the chromatographic separation, the columns and LC conditions that were used were different for the polar metabolite fraction versus the non-polar lipid fraction. For the polar metabolite fraction, 5 μL injections on a Kinetex Polar C18 column (100 mm × 2.1 mm, 2.6 μm) from Phenomenex (Torrance, CA, USA) at a flow rate of 0.3 mL/min at 40 °C were done. Mobile phase A consisted of 0.1% formic acid in water, and mobile phase B was 0.1% formic acid in acetonitrile. The gradient was as follows: 5% B at 0–2 min, 77% B at 8 min, 95% B at 12–14 min, and 5% B at 14.2–18 min. As for the non-polar lipid fractions, chromatographic separations of 5 μL injections were performed on an XSelect Acquity CSH C18 column (100 mm × 2.1 mm, 3.5 μm) from Waters Corporation (Milford, MA, USA) at a flow rate of 0.35 mL/min at 40 °C. Mobile phase A consisted of 60:40 acetonitrile/water with 10mM ammonium formate and 0.1% formic acid, and mobile phase B consisted of 90:10 isopropanol/acetonitrile with 10mM ammonium formate and 0.1% formic acid. The gradient was as follows: 10% B at 0–2 min, 40% B at 4.5 min, 79% B at 9.5 min, 95% B at 18.5–21.5 min, and 5% B at 21.6–25.1 min.

For the MS data acquisition after chromatographic separation, compounds were ionised with both polarities (i.e., positive and negative) using Dual Agilent Jet Stream Electrospray Ionisation with the following source conditions for the Q-TOF mass spectrometer system: a positive capillary voltage of 4 kV in positive ion mode, negative capillary voltage of 3.5 kV in negative ion mode, drying gas flow of 10 L/min, and the gas temperature of 320 °C. The nebuliser pressure was set at 35 psi. The Fragmentor voltage of the method was set at 75 V. Data was acquired over a mass range of 50–1000 *m*/*z*. Agilent Masshunter Auto MS/MS mode were used for MS/MS data acquisition. A collision energy of 10, 20, and 40 eV was applied for Data Dependent Acquisition (DDA) for both positive and negative modes, with an acquisition rate of 4 spectra/s. A maximum of 10 precursors were selected for each MS cycle for MS/MS acquisition. ‘Iterative’ mode was applied, in which MS precursors selected for a particular run were not selected for subsequent duplicate MS/MS runs.

After data acquisition, raw data files were converted to the mzXML format with the open-source ProteoWizard software [20], before processing with the XCMS Online program (https://xcmsonline.scripps.edu (accessed on 30 June 2022)). In XCMS, blanks, QC and plasma samples were uploaded for pre-treatment, including procedures such as peak picking and grouping, retention time correction, peak alignment, and annotation of isotopes and adducts. Specifically, we followed and adapted the recommended parameters for the UPLC/QTOF mode for untargeted metabolomics works available in XCMS to ensure accuracy and consistency of the integration of peaks. For feature detection, 15 ppm was set as the maximal tolerated *m*/*z* deviation in consecutive scans, with minimum and maximum peak widths of chromatographic peaks being 5 and 20 s respectively. Retention time was corrected by using 1 *m*/*z* step size for profile generation from the raw data files using the ‘obiwarp’ method. Alignment of the peaks across samples was done with the following parameters: (a) 5 s allowance for retention time deviations and (b) half of the samples in at least one sample group must have the peak for it to be valid. The adducts included during the annotation step include [M+H]^+^ and [M+Na]^+^ for the positive mode, and [M-H]^−^ and [M+FA-H]^−^, which are potentially key ESI adducts formed based on the mobile phases used. After checking for the accurate and consistent integration of peaks across samples, information containing the sample retention times, *m*/*z*, peak abundance and isotope ions was downloaded from XCMS to be used for further data clean-up. Isotope ions were filtered and only the main isotope was retained for each feature.

#### 2.2.4. Identification of Highly Contributing Features and Pathway Analysis

Identification of highly contributing features for the untargeted metabolomics and lipidomics datasets was based on the criteria considering both univariate and multivariate results obtained from MetaboAnalyst (http://www.metaboanalyst.ca (accessed on 30 June 2022)). Univariate tests for comparing mean peak areas between subject groups were done using the Mann–Whitney test. *p*-values obtained were false-discovery rate (FDR)-adjusted. Principal component analysis (PCA) and projection to latent structures–discriminant analysis (PLS–DA) were then performed. The PLS–DA model was validated with 10-fold cross-validation and a 100-iteration permutation test. The criteria for selecting important features for identification were as follows: a variable importance in projection (VIP) score > 2.0 (includes metabolites/lipids identified in both principal components (PC) 1 and 2) and a univariate test *p*-value of <0.05 with fold-change >1.5 or <0.67.

For those significant features based on the aforementioned criteria, a further structural identification step was required. For that purpose, a 3-step process was employed for putative identification. Firstly, matching of accurate *m*/*z* (accuracy threshold = 10 ppm) to online databases was done. The Human Metabolome Database (HMDB; http://hmdb.ca (accessed on 30 June 2022)), was used for the majority of the matches and class identifications. This was supplemented with matches using the METLIN database (https://metlin.scripps.edu (accessed on 30 June 2022)). Secondly, MS/MS spectra were obtained either from DDA-based results or additional MS/MS runs, and final identities were determined by comparing our MS/MS spectra with the in silico fragmentation spectra of potential molecular identities shortlisted from the first step. Thirdly, a final validation was done by checking that the retention time was reasonable based on the polarity of a proposed structure. As such, a level 2 metabolite identification confidence level may be achieved, according to the Metabolite Standards Initiative [21]. For features with no matching MS/MS spectra, they were grouped under the level 4 identification level, and are left out from network analysis due to insufficient molecular information.

### 2.3. Glycomics and Metallomics Analysis and Data Acquisition

N-glycan extracts and elemental digestates from blood plasma samples were obtained as described in our previous works [10,11], and also presented in the “Supplementary Methods” section found within the Supplementary Material file available online. Briefly, 2 μL blood plasma was treated with PNGase F and digestion buffer for the release of N-glycans. Before instrumental analysis, the N-glycans were reduced with 2-picolane borane and labelled with a fluorescence tag (8-aminopyrene-1,3,6-trisulfonic acid (APTS)). The APTS-labelled glycans were then purified via the use of a magnetic stand-enabled solid phase extraction step. As for the elemental digestates, briefly, equi-volumes of ultrapure nitric acid and ultrapure water were added to 80 μL blood plasma. Acid digestion proceeded on a hotplate at 98 °C for 2 h. The glycan extracts and elemental digestates were then subsequently diluted to suitable levels and analysed by capillary electrophoresis-laser-induced fluorescence (CE-LIF) and inductively coupled plasma-mass spectrometry (ICP-MS) respectively, again as described in our previous works [10,11]. For the glycomics dataset, peak areas were obtained for each N-glycan variable, while concentrations were obtained for the metallomic variables via the building of external calibration curves with appropriate standards.

### 2.4. Data Processing

Data pre-processing was done to filter out low-quality metabolite/lipid/glycan/element peaks/concentrations via the following criteria: (1) peaks with less than 3-fold average intensity in the samples compared to the blanks; (2) peaks present in less than 75% of the samples in either patient/control group; (3) relative standard deviations (RSD) of intensities more than 20% in pooled QC samples (except for RSD threshold of 30% for the untargeted metabolomics portion). After filtering, any missing values were replaced with half the minimum value of each respective feature.

For the glycomics dataset, normalisation of peak areas was done with respect to the sum of peak areas as per the glycomics convention. On the other hand, for the metabolomics and lipidomics datasets, normalisation was done with respect to the QC samples by the internal standard [22]. No normalisation was done for the metallomics dataset as accurate concentrations were measured.

To prepare the four omics datasets for cross-omics multivariate analysis, log-transformation and scaling via Pareto scaling were done as appropriate.

### 2.5. Statistical Analysis and Multi-Omics Integrative Analysis

All statistical analyses were conducted in *R* (version 4.04, R Core Team, Vienna, Austria) [23]. The normality of all metabolites, lipids, and glycan features was first assessed via the Kolmorov–Smirnov test. Since non-normal distributions (*p* < 0.05 based on the Kolmorov–Smirnov test) were found with some of the features, non-parametric tests were done subsequently. Baseline characteristics among patients and controls were compared using the Mann–Whitney test for continuous variables, and Fisher’s Exact test for categorical variables. Differences in plasma levels of the various omics features between patient groups were also assessed using the Mann–Whitney test (with a Benjamini–Hochberg multiplicity correction if comparisons between multiple patient groups were endeavoured).

For the multi-omics analysis, the ‘mixOmics’ package (version 6.0.0) was used and implemented in R [24]. The DIABLO framework from the ‘mixOmics’ package was used to integrate the metabolomics and glycomics datasets via block-PLS-DA based on sparse generalised canonical correlation analysis (CCA). The PLS-DA model was tuned and performed with 10-fold validation and using centroid distance for estimating the error rate. The visualisation of the relationships between variables from all four omics layers (based on results from the DIABLO framework’s block-PLS-DA analysis) was done by plotting relevant correlation plots and relevance network maps using the ‘mixOmics’ package.

Classification modelling was done on MetaboAnalyst (http://www.metaboanalyst.ca (accessed on 30 June 2022)). The receiving operating characteristic (ROC) curve for biomarker identification and performance evaluation was generated based on random forest classification modelling with repeated random sub-sampling cross-validation (30 iterations; each iteration uses 2/3 samples for feature selection and model training, and the remaining 1/3 for testing).

## 3. Results and Discussion

### 3.1. Demographic Information and Baseline Characteristics

Table 1 summarises the baseline characteristics such as demographic information (i.e., age, gender, race), medical history related to the disease, as well as traditional cardiovascular risk factors and predictors of AMI and adverse events of the study populations. The following discussion regarding the demographic information and baseline characteristics of the samples had been mentioned in our previous works [10,11]. They are briefly reiterated in this work. Firstly, we observed no significant differences in the distributions of patient ages between the AMI and healthy groups, as they were age-matched in the study. On the other hand, other demographic characteristics such as gender, smoking status, and race were found to be significantly different between the two groups (*p* < 0.0001).

Secondly, with reference to Table 1, we confirmed that the medical history for CHD was also closely matched between AMI and healthy groups, with the only exception being dyslipidaemia (*p* < 0.001). Differences in high-sensitive Troponin T (hsTnT) and N-terminal-pro hormone brain natriuretic peptides (NTproBNP) levels were found to be highly significant between patient groups (*p* < 0.0001). Nonetheless, this observation was expected as they are biomarkers of AMI, and raised troponin was also an inclusion criterion for the AMI cases in this study. As for other traditional cardiovascular risk factors, several of them (diastolic blood pressure (DBP), systolic blood pressure (SBP), high-density lipoprotein-cholesterol (HDL-C), white blood cell count (WBC), and creatinine levels) are also statistically significant variables (*p* < 0.0001), which again was expected due to their associations with CHD.

### 3.2. Analytical Validation for Untargeted Metabolomics and Lipidomics Analysis

Prior to the formal analysis of study samples, the combination of the extraction method with our LC-QTOF analytical workflow for metabolomics and lipidomics analysis was validated by assessing instrumental analytical precision, and inter-day method repeatability. As for the glycomics and metallomics analytical workflows, again, they were validated and relevant figures-of-merit had been presented in our previous works [10,11].

Firstly, for checking the instrument’s analytical precision, especially necessitated by LC-MS’s known signal drift and batch issues, a single extract was injected 10 times in succession, in both positive and negative modes. RSDs of 7.9% (positive mode) and 5.5% (negative mode) were obtained for the 10 consecutive injections, indicating satisfactory instrument precision. As such, for the actual analysis of study samples, the following run conditions were set: (1) 10 consecutive injections of a QC sample prior to sample injections for conditioning, and (2) one QC injection between every 10 sample injections for quality control.

For assessing inter-day repeatability/precision, three independent sets of extractions were done using the same pooled plasma sample, and they were analysed over different days to evaluate any significant batch effects and uncertainties in the overall workflow. Extraction and analysis of both the metabolite and lipid fractions were done in both positive and negative modes (four sets in total). A threshold of up to 30% RSD was used for reference, as it is commonly accepted as a standard when filtering samples based on QC in metabolomics works [25]. For the metabolite fraction, 58.0% of peaks from the positive mode and 88.0% of total peaks from the negative mode had an RSD < 20%. As for the lipid fraction, median inter-day RSDs of 14.0% (positive mode) and 12.9% (negative mode) were achieved. Additionally, 79.0% of the total peaks from the positive mode and 86.2% of total peaks from the negative mode had RSDs of <20%, indicating great precision, which is beyond comparable with the standard. While the results for the polar metabolite fraction in the positive mode are less desirable, the overall results for all four sets gave satisfactory intra-day precisions. Based on these results, we thus also set the RSD threshold during QC filtering of the actual sample run to be at 30% for the metabolomics analysis and 20% for the lipidomics portion, i.e., features with more than 20% RSD in QC samples for the lipidomics portion were removed.

### 3.3. Generation of Untargeted Lipidomics and Metabolomics Datasets and Identification of Significant Features

Firstly, the human plasma untargeted metabolomics and lipidomics profiles were generated as described, based on the Matyash extraction method, and according to the run/QC conditions ascertained in the previous section. Metabolite/lipid features extracted using XCMS were first defined by their LC retention time and accurate mass-to-charge ratios (*m*/*z*), as their structural identities were unknown at this point. For the metabolite fraction, 6361 and 4228 features were extracted from the positive and negative modes’ results, respectively. For the lipid fraction, 15,968 and 3366 features were extracted from XCMS from the positive and negative modes’ results, respectively.

Secondly, data clean-up such as the removal of features with RSDs of >20 or 30% in the QC, as well as filtering after data normalisation, was performed as described in the methods Section 2.4. Based on these data processing steps, the following number of features were left for further statistical filtering: 319 (positive mode) and 1420 (negative mode) for the metabolite fraction, and 3292 (positive mode) and 973 (negative mode) for the lipid fraction.

**Figure 1:**
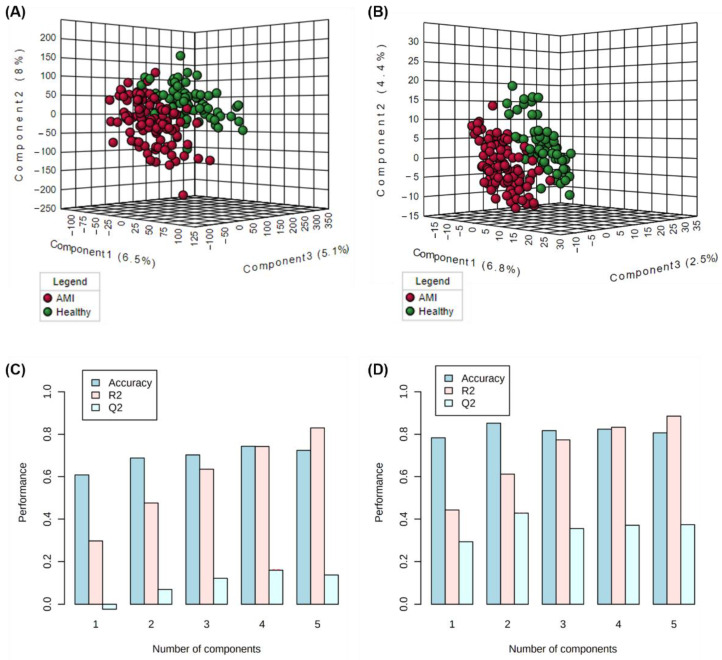
PLS-DA score plots for the visualisation of clustering of AMI versus healthy samples based on (**A**) lipidomics and (**B**) metabolomics analysis, and the corresponding model performance measures across the number of components generated from (**C**) metabolomics and (**D**) lipidomics analysis.

Thirdly, these validated and “cleaned-up” datasets were then further filtered based on statistical criteria as described in the methods Section 2.2.4. This process served to generate a focused list of highly contributing features in differentiating AMI and healthy patients, for further structural identification. A total of 40 metabolomic and 99 lipidomic significant (*p* < 0.05) features were filtered. Since one of the criteria for the filtering of significant features is based on the VIP score generated from PLS-DA modelling, the models’ performances were checked. Figure 1A,B show the constructed three-dimensional PLS-DA models (with 10-fold cross-validation) for the metabolomics and lipidomics portions, respectively. Figure 1C,D illustrate the various performance measures (accuracy, R^2^, and Q^2^) against the number of components used in the PLS-DA models. For the metabolomics analysis, the cumulated R^2^ and Q^2^ values at the optimal number of components were 0.79 and 0.22 for metabolomics, and 0.61 and 0.43 for the lipidomics analysis. The moderately high R^2^ and closeness of Q^2^ to R^2^ values for the lipidomics analysis demonstrate good predictive relevance and validity, based on PLS model performance standards [26]. On the other hand, for the metabolomics portion, there may be possible overfitting, and discretion may be advised when interpreting results generated from PLS-DA-based models for our metabolomics dataset.

Finally, a three-step structural identification process as described in Section 2.2.4 was applied to annotate the identities of the 40 metabolomic and 98 lipidomic features. In this untargeted metabolomics/lipidomics study, a Level 2 metabolite identification confidence level was achieved for most of the significant features (according to the Metabolite Standards Initiative) [21]. Unfortunately, there was difficulty identifying potential structures based on an accurate *m*/*z* and in the matching of the MS/MS spectral patterns for multiple features. Subsequent analyses and interpretations were thus limited to what could be surmised from the identified features.

As such, a total of 76 (27 metabolomic and 48 lipidomic) features could be successfully annotated, and their most pertinent details are given in Table 2 (metabolomics) and Table 3 (lipidomics). As for those unannotated features, they continued to be included in classification modelling. LC–MS results and statistical information on these unannotated significant features are given in Table S1 (metabolomics) and Table S2 (lipidomics) in the Supplementary File, together with the additional structural information and the VIP scores of the identified features. However, they were excluded from multi-omics integration due to insufficient information.

### 3.4. Single-Omics Evaluation of Metabolomics and Lipidomics Datasets

Based on the metabolomics and lipidomics profiles of significantly altered features, various biological insights may already be discerned. Since numerous similar metabolomics or lipidomics studies have already been attempted on CHD populations [27,28,29,30,31,32,33,34,35,36,37,38,39,40,41,42,43], this work will only present the “single-omics” observations briefly, to serve as a basis for comparison with the extended capacities a multi-omics integration may offer.

First, the metabolite/lipid classes that were significantly altered in AMI were assessed. With reference to Table 2, it is shown that there is a spread of metabolite classes involved with AMI risk. They include semi-polar compounds such as purine nucleosides and indole/derivatives, to non-polar compounds such as steroids/derivatives (overlap with lipidomics’ coverage). We note that a few of the metabolites found at higher levels in AMI patients do not seem to be endogenous/non-naturally occurring in the human body. They include (1) perfluorododecanoic acid, a polyfluoroalkyl chemical found in stain-resistant furniture, grease-resistant paper, kitchen wares, etc., whereby its exposure and accumulation in human serum has been reported to be associated with cardiovascular risk and specifically angina pectoris (chest pain) [44], and (2) carbosulfan, a pesticide, and a derivative of carbofuran, the exposure to which has several reported associations with CVD risk and AMI [45]. This untargeted metabolomics study thus augments the evidence pointing at such chemical exposures as potential CVD risk factors.

**Figure 2:**
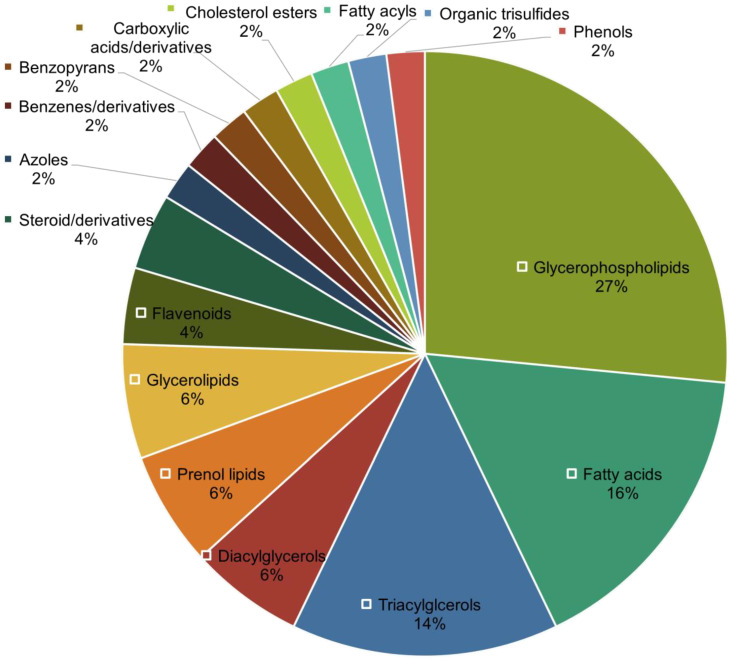
Distribution of lipid classes of annotated significant lipidomic features.

Second, unlike the metabolomics portion, great coverage for lipidomics was observed (Table 3), with significant lipid features constituting five out of the eight main lipid categories defined by LIPID MAPS [46]. We thus further investigated the distribution of lipid classes that were significantly altered (*p* < 0.05) in AMI. The distribution of lipid classes was plotted as a pie chart as shown in Figure 2. Based on Figure 2, glycerophospholipids (27%), fatty acids (16%), and triacylglycerols (14%) are the top three abundant lipid classes that were altered in AMI. As shown in Table 3, these lipid classes display distinct regulation patterns: glycerophospholipids were generally downregulated in AMI patients, all detected fatty acids were downregulated, while all triacylglycerols (as well as most diacylglycerols) were unanimously upregulated in AMI.

As for the down-regulation trend found in glycerophospholipid metabolism, this has already been reported to play a prominent role in CHD progression recently [47]. According to Chen et al., plasmalogens (including various phosphatidylcholines (PC) and phosphatidylethanolamines (PE) identified in our work and classified under glycerophospholipids in Table 3) were proposed to be protective against atherosclerosis [47]. Moreover, since plasmalogens are also notably more susceptible to oxidation under oxidative stress, low levels of such glycerophospholipids can be considered as a biomarker of oxidative stress and the negative actions of reactive oxygen species, which drive or can be driven by disease progression [48,49]. Additionally, glycerophospholipids have been postulated to be possible inflammatory mediators as glycerophospholipid metabolic pathway was associated with low-grade inflammatory states and general systemic-immune inflammatory states [50]. This link suggests the potential and value of integrating this lipidomics dataset with glycomics, to identify interactive metabolic pathways commonly associated with inflammation.

As for the simultaneous down-regulation of fatty acids and up-regulation of di- and tri-acylglycerols, this can be explained by the imbalance between fatty acid uptake and oxidation in AMI patients. This is since these two categories of lipids share a dynamic balance, as di- and triacylglycerols are lipid intermediates (alongside phospholipids and sphingolipids) that will accumulate within the myocardium when fatty acid oxidation (FAO) is unable to match the fatty acid delivered to the heart [51,52]. Overall, this simultaneous dysregulation of fatty acids and di-/triacylglycerols (as well as up-regulation of other lipid intermediates attributed to the dysfunction of FAO) served as additional evidence for the ongoing discussion on “lipotoxicity” in the human heart. As summarised by a key review by Schulze et al., there have been various supporting reports on the role of intracellular lipid accumulation leading to chronic states with ATP production dysfunction and energy depletion of the failing myocardium [53]. Such reports range from studies of animal models, studies of patients with inborn errors of FAO who develop cardiac abnormalities (e.g., sudden cardiac death, cardiac/skeletal myopathies, insulin resistance), and observational studies reporting the association of cardiac lipid accumulation with obesity and/or metabolic cardiovascular complications (e.g., diabetes mellitus), as well as more advanced imaging studies showing increased intramyocardial lipid content in patients with heart failure [53]. However, while it seems apparent that lipid accumulation is linked to the failing myocardium and thereby also AMI onset and progression, what initiates or drives it is still unclear (i.e., whether it is due to elevated fatty acid uptake, elevated di-/triacylglycerol synthesis, impaired degradation of lipids, or combinations of the above) [53].

### 3.5. Multi-Omics Integration and Analysis of Cross-Omics Relationships

**Figure 3:**
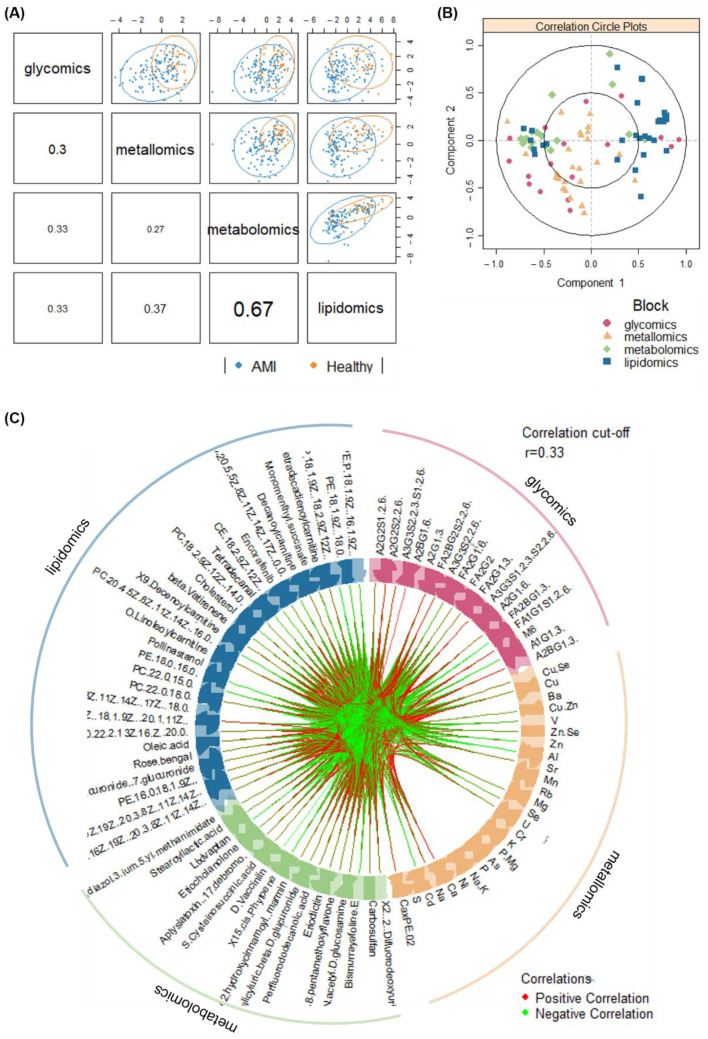
Visualisation of correlations amongst omics datasets via (**A**) sample scatterplot displaying the first component in each omics block (upper diagonal) and Pearson correlation between each component (lower diagonal), (**B**) correlation circle plot representing feature contributions from each omics block, and (**C**) circos plot showing the correlations (*r* > 0.33) between omics features as indicated by the red (positive correlation) and green (negative correlation) links.

Next, the annotated metabolomic (number of features, *n* = 27) and lipidomic (*n* = 48) profiles, along with the glycomic (*n* = 37; [10]) and metallomic (*n* = 30; [11]) profiles of the same set of AMI and healthy samples were integrated and analysed. The integration method used here works by identifying a linear combination of maximally correlated features across the four omics datasets, via utilising both block-PLS-DAs based on sparse generalised canonical correlation analysis [54]. A series of correlation plots (Figure 3) display the overall correlations amongst the omics blocks.

Primarily, Figure 3A showed that the latent components of each omics block were highly correlated between metabolomics and lipidomics (*r* = 0.67), and were moderately correlated between metallomics and lipidomics (*r* = 0.37), between glycomics and lipidomics (*r* = 0.33), and between glycomics and lipidomics (*r* = 0.33). This highlights the ability in modelling satisfactory agreement between the datasets. The clustering of the AMI and healthy samples, while not distinct, are inhomogeneous and thus preliminarily demonstrates the ability of the integrated omics models to discriminate the outcome of interest as well. However, low correlations (*r* < 0.33) were observed between glycomics and metallomics, and between glycomics and metabolomics data. Nevertheless, beyond general agreement among the various omics blocks in disease classification, further assessment for specific components highlights the correlations between individual features from each omics block (Figure 3B). Additionally, the circos plot (Figure 3C) displays the significantly contributing features across all four omics blocks in a circle, with links between each omics feature indicating at least a moderate correlation (*r* cut-off = 0.33; *p*-value cut-off at 0.05). The density of both red (positive correlation) and green (negative correlation) links in Figure 3C demonstrates the high-volume interactions amongst biomolecules from all four omics layers.

**Figure 4:**
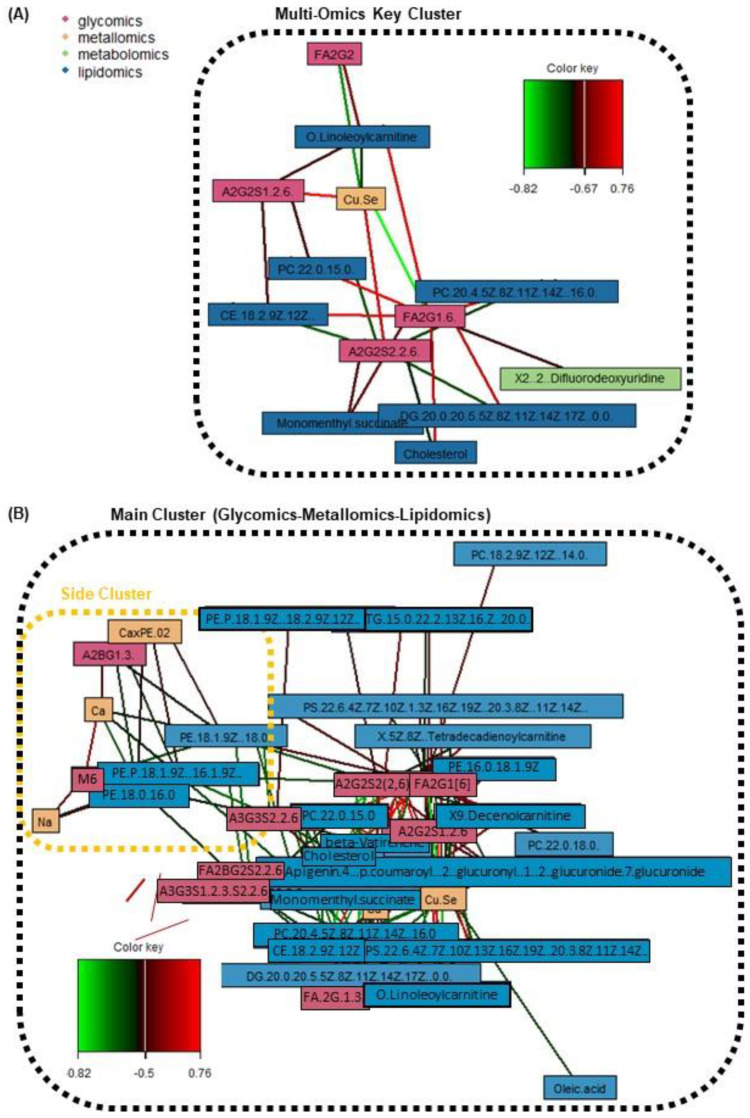
Relevance network plots of significant omics features from the discrimination of AMI vs. healthy patients, displaying (**A**) key cluster amongst all four omics with strong correlations between features (*r* ≥ 0.67; *p*-value < 0.05), and (**B**) tri-omics (glycomics + metallomics + lipidomics) correlations (*r* ≥ 0.5; *p*-value < 0.05). The color key indicates the correlation coefficient values annotated by the connection lines between variables. Red colored connection lines denote positive correlations, while green colored connection lines denote negative correlations between variables. The intensity of the colors is scaled according to the magnitude of the correlation coefficient values.

**Figure 5:**
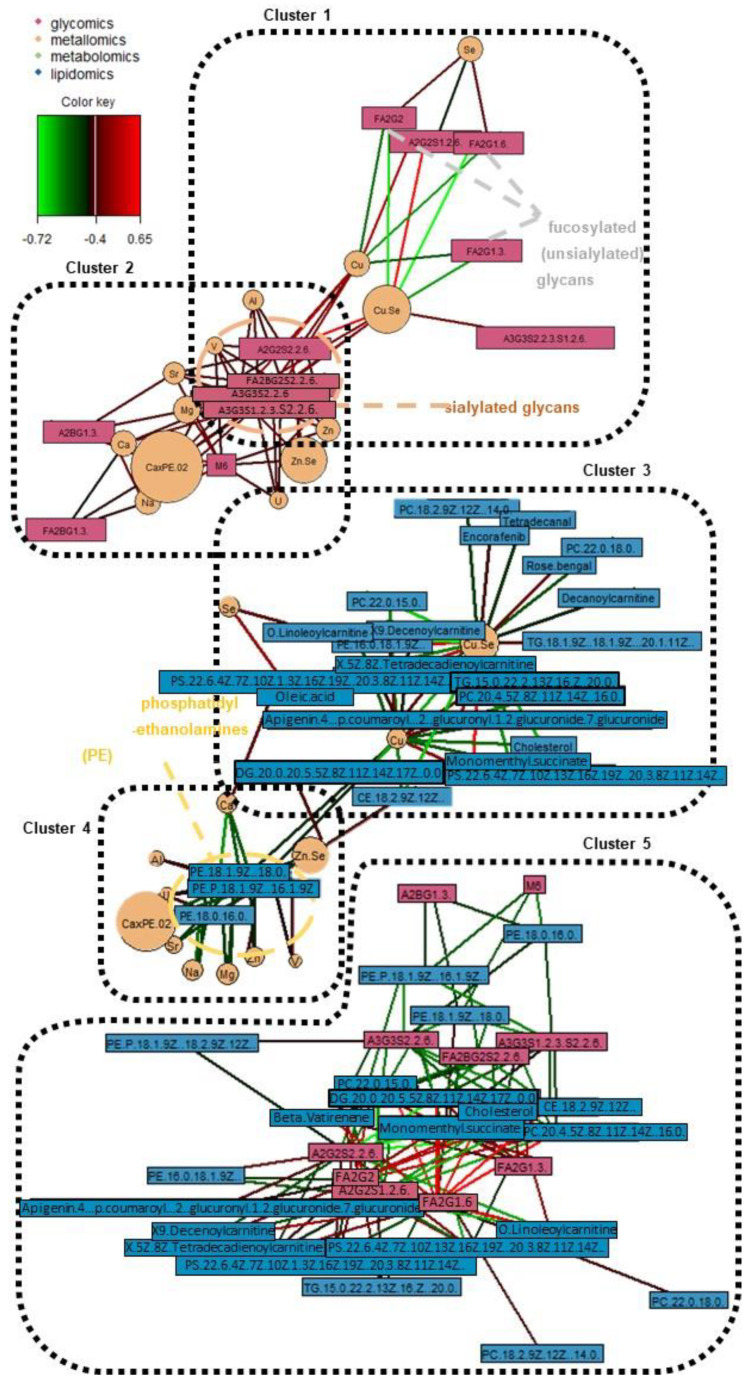
Relevance networks of significant omics features across various bi-omics combinations (all *r* ≥ 0.4; *p*-value < 0.05). The color key indicates the correlation coefficient values annotated by the connection lines between variables. Red colored connection lines denote positive correlations, while green colored connection lines denote negative correlations between variables. The intensity of the colors is scaled according to the magnitude of the correlation coefficient values.

Next, through the generation of a series of relevance network plots amongst various omics combinations, various cross-omics clusters of interest have been identified. Figures S1–S9 in the Supplementary File display the individual relevance network plots (including one tetra-omics network, two tri-omics networks, and all six combinations of the bi-omics networks; a high correlation cut-off at *r* ≥ 0.5 and *p*-value < 0.05). On the other hand, Figure 4 and Figure 5 display the main relevance network plots, which reveal pertinent clusters.

Firstly, with reference to Figure 4A which shows the key cluster (*r* ≥ 0.67) from all four omics blocks, it mainly revolves around the FA2G1[6] glycan and A2G2S2(2,6) glycan. FA2G1[6] is a core-fucosylated and unsialylated glycan, while A2S2S2(2,6) is a fully-sialylated biantennary and noncore-fucosylated glycan. These glycans, which are abundantly found on the Fc region of human IgG, are both also correlated with the Cu/Se ratio, and lipids of various classes (acyl carnitine, glycerophospholipid, diacylglycerol, cholesterol and cholesterol ester). However, it is interesting to note that the directions of correlations are opposite for the two glycans—FA2G1[6] is positively correlated with various lipids and negatively correlated with the Cu/Se ratio, while A2G2S2(2,6) is negatively correlated with various lipids but positively correlated with the Cu/Se ratio. The other two glycans found in the key cluster (FA2G2 and A2G2S1(2,6)) follow the same trends in terms of glycosylation traits versus the correlation direction with both lipids and the Cu/Se ratio. In fact, when looking at Figure 4B and specific bi-omics (glycomics–lipidomics and glycomics–metallomics) relevance networks at *r* = 0.4 cut-offs in Figure 5’s Clusters 1, 2, and 5, the aforementioned correlation trend is consistent and even more apparent. This reveals intriguing insights regarding the immunoregulatory capacity of IgG in AMI and its possible interplay with lipid membrane domains.

We also looked at how the various detected glycans are linked to IgG–FcγR functions, as well as how IgG–FcγR interactions could be inter-regulated by lipid metabolism. Fc region glycans can directly influence the affinity of IgGs to FcγRs and thereby change their ability to recruit immune effector cells to activate distinct immunomodulatory pathways, either by changing the conformation of the Fc region or via glycan–glycan interactions [55,56]. For example, N-glycans with core fucose or terminal sialic acid present reduce the affinity of IgGs to Fcγ receptors in general [55], but specifically, unsialylated IgGs were reported to primarily interact with type I Fc receptors, which include all FcγRs. On the other hand, sialylated IgGs interacted primarily with type II FcγRs, which are mainly expressed in dendritic cells (DC) [55,57]. Next, lipid metabolism, and thereby lipid content/composition in immune cells, may be inter-regulated by IgG–FcγR interactions [58]. Now, as seen from the correlation trends with the various lipids seen in Figure 4, most of the lipids (11 lipids) linked with the glycans associated with the IgG–FcγR immune complex are glycerophospholipids (one of the three main lipid classes composing plasma membranes). Furthermore, it has been previously reported that pro-inflammatory activated lipid-rich human monocyte-derived DCs, with their remodelled plasma phospholipid and cholesterol content, might be involved in atherosclerosis [59]. All of these collaboratively suggest that our previous postulation regarding AMI atherosclerosis involving a switch from a pro-inflammatory to an anti-inflammatory state could have also been modulated by lipid metabolism. On the other hand, we postulate that it could also have simultaneously triggered a change in plasma membrane composition to elicit desired immunoregulatory responses as a response to atherosclerotic onset or progression. The involvement of Cu/Se ratio in this equation remains unclear, although Se has been shown to regulate murine lipid levels via restoring lipid peroxidation and ameliorating disruptions of membrane dynamics [60]. It is likely that Cu and Se are indirectly involved in this relevance network due to their strong but general role in inflammation and immunity [61]. This is further shown as we look at Figure 5’s bi-omics (metallomics–lipidomics) Cluster 3, where Se is only positively correlated with a fatty acid and its acylcarnitine (oleic acid and o-linoleoylcarnitine) with *r* ≥ 0.4. This also highlights the need to investigate the relationship between copper and lipids, which has 15 links *r* ≥ 0.4, yet lacks evidence of their interaction in CVD pathophysiology.

Beyond the key cluster identified in the multi-omics network in Figure 4A, a side cluster was also revealed when the correlation cut-off was lowered to *r =* 0.5 (Figure 4B). This side cluster revolves around Ca and its interactions with two unsialylated and non-fucosylated glycans (A2BG1[3] and M6) and three phosphatidylethanolamine (PE) lipids under the glycerophospholipid class. It is noted that Ca’s negative correlation with the PEs seemed to be specific, as correlations with other types of lipids, glycerophospholipids or otherwise, were absent at this correlation cut-off. Thus, while Ca ions have been reported to interact with lipid membranes as a whole (as evidenced by the presence of Ca binding sites in lipid bilayers and changes in membrane bilayer structures with Ca binding, etc.) [62], the lack of Ca–PC and Ca–phosphatidylserine (PS) correlations suggest a different and more specific mode of interaction with the PEs. Here, our evidence (along with the correlations with Na and Ca×P products) points towards the role of the sarcoplasmic reticulum Ca–ATPase (SRCA) in heart disease. The PE headgroups had been reported to modulate SRCA function by facilitating “dynamic structural changes involving the phosphorylation domain, which enhances the catalytic function of Ca–ATPase” [63]. This explains the link between the PEs with Ca, and thereby also possibly Na, since Na together with Na/K–ATPase play interactive roles in the regulation of intracellular Ca as well as Ca stores at the SRCA [64]. All of these have direct consequences on cardiac myocyte functions and are linked with heart contractions [64]. Since SRCA is a potent therapeutic target for CVD [65,66], our work suggests to also scrutinise specific intermolecular interactions that result from the phosphoethanolamine moiety of PEs. Moreover, it is also interesting to see from Figure 5 Cluster 4, that PEs not just have strong correlations with Ca and Na, but also moderate correlations (*r* ≥ 0.4) with many other elements as well (e.g., essential elements such as Al, Cu, Mg, and Zn). It thus highlights the multi-faceted interactions that PEs have with such essential elements, and their potential importance in phospholipid homeostasis in the cardiovascular system [67,68]. Lastly, further studies should also investigate how a terminal GlcNAc glycan and a high-mannose glycan as identified in this work may be involved, e.g., whether inhibition of the ATPases impairs N-glycan expression/function [69], or vice versa [70].

### 3.6. Classification Modelling and Performance of the Multi-Omics Model

**Figure 6:**
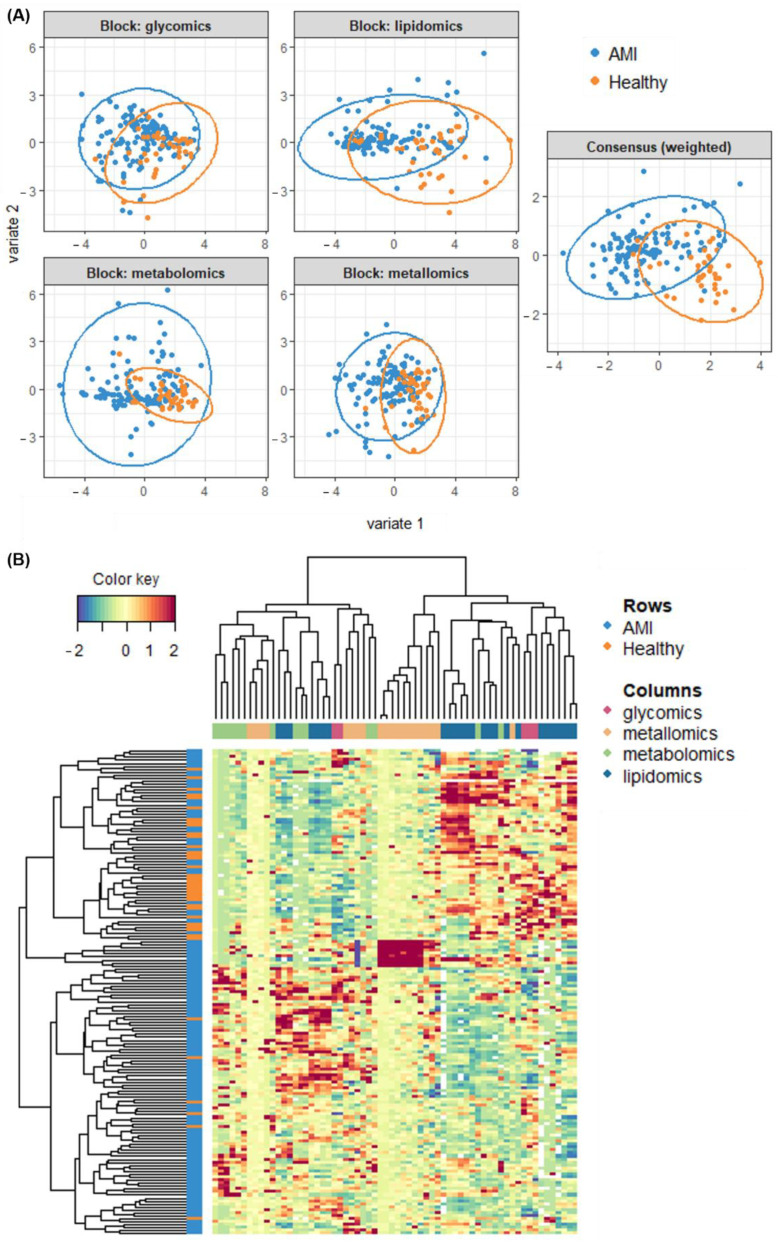
Classification performance of glycomics, metallomics, metabolomics and lipidomics datasets as shown from (**A**) block-PLS-DA analysis and comparison of individual blocks with a consensus multi-omics model, and (**B**) hierarchical cluster analysis of the multi-omics dataset.

As a culmination of all four omics studies, the performance of a multi-omics classifier in discriminating between AMI and healthy was compared with that of the individual omics. First, for quick visualisation of the classification performances, PLS-DA modelling was applied to depict the classifiers’ ability to cluster/separate AMI versus controls. For the multi-omics classifier, it was built based on the block-PLS-DA analysis described earlier. As shown in Figure 6A, the ability to cluster/separate AMI and healthy samples via the consensus multi-omics classifier is seemingly better than the individual PLS-DA models.

Secondly, besides the quick visualisation based on supervised PLS-DA models, an unsupervised method was also applied to observe if the discrimination between AMI and controls was indeed the main source of variability measured in omics variables. For that purpose, hierarchical cluster analysis was done. As shown in Figure 6B, the use of all four omics datasets was able to render the clustering of AMI from healthy relatively well. This can be seen from the grouping of the blue rows (AMI) mostly at the bottom, while the orange rows (controls) are mostly on top. Beyond that, the intermixing of features from the four omics layers could also be observed from the top dendrogram in Figure 6B. This was shown from the interspersed spread of the colored columns (showing features from different omics layers). The significance of this observation is that it possibly denotes an interactive relationship among the four omics in AMI discrimination.

Thirdly, to more objectively assess the differences in performance between individual and multi-omics classifiers, AUC_ROC_s were generated. In this case, random forest models were chosen as the standard modelling technique for comparison purposes, to account for any nonlinear interactions across the omics layers. As a note, random forest modelling was also chosen instead of PLS-DA modelling as PLS-DA is a linear-based method and may not be sensitive to the nonlinear cross-omics relationships. Again, the random forest modelling was done with repeated random sub-sampling cross-validation (30 iterations; each iteration uses two-thirds of the samples for feature selection and model training, and the remaining one-third for testing). No external validation was done. The results are summarised in Table 4 below. The performance of the multi-omics classifier (AUC_ROC_ = 0.953) was higher than all the individual omics classifiers. The multi-omics classifier’s lower confidence limit (CI 0.911–0.987) was also even higher than all the upper confidence limits of the individual classifiers, except for metabolomics. This exemplifies the synergistic effect when these four omics datasets were cohesively used to build a disease classification model. Although, there is one caveat here: the observation that the multi-omics classifier achieving a higher AUC_ROC_ value is not unexpected mathematically. The most important question remains to be whether the multi-omics classifier’s performance can be reproducible when tested in a large and independent cohort of AMI patients. This is the litmus test.

As a whole, despite the limitation that an external validation was not achieved in this portion, the potential of the multi-omics classifier, having synergistically enhanced performance, cannot be denied.

### 3.7. Limitations and Further Work

One of the biggest analytical challenges still pertains to the coverage in metabolomics analyses. As such, for future large-scale multi-omics studies, researchers should consider performing targeted metabolomics with a defined list of pathways and metabolites of interest [71], or to attempt, as comprehensively as possible, an untargeted metabolomics study by perhaps performing both GC–MS and LC–MS analyses for covering both volatile and non-volatiles [71], as well as running LC–MS with both normal and reverse-phased columns (e.g., HILIC + T3 + C8 combination) [72], after testing at different mobile phase pH conditions (acidic, neutral, basic) for optimal/complementary selectivity [73].

Secondly, in this study, only age was matched between the case and control groups. However, other demographic characteristics (gender, race, smoking status), as well as cardiovascular risk factors are also important factors that may be translated to measurable differences in omics profiles as well. Unfortunately, our study size was severely limited by patients’ recruitment in local hospitals. Thus, we could only match the cases and controls based on age. Moreover, the inequity in distributions was further compounded by the fact that AMI patients are usually males. Additionally, adjustment for confounding factors by statistical analysis cannot completely eliminate the effect of mismatching between the two groups on the results. Therefore, in future work, sensitivity analyses (e.g., by removing some female controls and checking if the results would be affected) should be attempted. Otherwise, studying larger cohorts with appropriate matching would be ideal.

Lastly, we also note the challenges pertaining to visualisation capacities in our work, which were limited by the functions achievable using the available open-sourced software/packages. It would be greatly beneficial for any such omics studies in the future to have further developments in bioinformatics tools to streamline and hasten the data processing, analysis, and visualisation works.

## 4. Conclusions

Overall, an integrative multi-omics study of omics beyond the central dogma was attempted on a cohort of AMI and healthy patients to reveal intriguing biological insights pertaining to the cross-omics mechanisms associated with AMI pathophysiology. They included elucidating the potential roles of glycerophospholipids in N-glycan-modulated IgG–FcγR’s immunoregulatory functions, the importance of SRCA in CVD, and the contributions of PEs for SRCA functions. Moreover, combining the four omics datasets synergistically enhanced the classifier performance in discriminating between AMI and healthy patients. All these discoveries were otherwise not attainable when these omics were singularly and independently analysed. The transitioning of a single omics to a multi-omics strategy is therefore supported by the work presented in this study.

It should also be noted that the significant contributions of the respective state-of-the-art analytical workflows employed in this work enabled efficient multi-omics research. We highlight that the CE-LIF-based glycomics workflow selected in this multi-omics study used only 2 μL of blood plasma, while the untargeted metabolomics and lipidomics datasets generated via simultaneous extraction used only 50 μL plasma. Furthermore, even though the metallomics workflow required acid digestion, which makes it hard to integrate with other omics extraction workflows, the glycomics and metabolomics/lipidomics extractions could potentially be unified. One of our recent works [74], which demonstrates the validity of a simultaneous polar metabolite and N-glycan extraction bi-omics workflow, supports such an outlook.

## Figures and Tables

**Table 1:** Demographic characteristics of the study populations. Significant *p*-values are underlined.

Characteristic	AMI (*n* = 101)	Control (*n* = 66)	*p*-Value
Demographic variables			
Age (Median (Min–Max))	57 (33–81)	54.5 (40–71)	2.7 × 10^−1^
Gender (Female) (%)	12.9	40.9	6.3 × 10^−4^
Smoking status (%)			
Non	37.6	90.9	
Current	35.6	3.0	
Ex	26.7	6.1	
Race (%)			1.0 × 10^−14^
Chinese	27.7	60.6	
Malay	21.8	9.1	
Indian	6.9	30.3	
Others	43.6	0.0	
Cardiovascular risk factors based on medical history			
Prior MI (%)	4.0	0.0	1.5 × 10^−1^
FH of CHD (%)	20.8	10.6	9.4 × 10^−2^
Diabetes (%)	20.8	12.1	2.1 × 10^−1^
Hypertension (%)	43.6	28.8	7.2 × 10^−2^
Dyslipidemia (%)	42.6	77.3	3.3 × 10^−5^
Other cardiovascular risk factors, predictors of AMI and adverse events (Median [Min–Max])			
DBP, mm Hg	83.50 (39–138)	74 (53–107)	1.6 × 10^−4^
SBP, mm Hg	140 (74–241)	127 (97–175)	8.2 × 10^−6^
Triglyceride, mmol/L	1.50 (0.35–5.58)	1.38 (0.37–4.37)	5.1 × 10^−1^
Cholesterol, mmol/L	5.01 (3.20–7.40)	5.32 (4.05–7.37)	1.5 × 10^−3^
HDL-C, mmol/L	1.03 (0.67–9.40)	1.34 (0.80–2.31)	6.6 × 10^−10^
LDL-C, mmol/L	3.20 (1.60–5.51)	3.27 (2.19–5.25)	1.1 × 10^−1^
WBC, ×10^9^/L	10.85 (4.40–25.90)	5.50 (2.60–9.0)	1.5 × 10^−20^
Platelet, ×10^9^/L	250.5 (134–649)	258 (165–599)	3.8 × 10^−1^
Creatinine, mmol/L	88.5 (51–142)	69 (42–97)	3.9 × 10^−13^
hsTnT, pg/mL	1726 (62–9819)	6 (3–18)	8.8 × 10^−28^
NTproBNP, pg/mL	718 (68–6819)	36 (7–150)	3.0 × 10^−27^

**Table 2:** List of annotated significantly altered metabolomic features.

Compound Class	Compound Name	RT|*m*/*z*	Regulation (Up/Down) ^a^	Fold-Change ^b^	*p*-Value ^c^
2-Arylbenzofuran flavonoids	Lixivaptan	6.83|472.29	DOWN	0.39	6.0 × 10^−5^
Alkyl halides	Perfluorododecanoic acid	6.41|613.36	UP	1.85	7.6 × 10^−9^
Benzene & substituted derivatives	1-Piperazinecarboxylic acid, 4-((3,4-dichlorophenyl)acetyl)-3-(1-pyrrolidinylmethyl)-, methyl ester, (3R)-	10.18|413.33	DOWN	0.64	2.1 × 10^−6^
	D-Vacciniin	4.57|283.08	UP	1.86	1.3 × 10^−5^
Carboxylic acids & derivatives	S-Cysteinosuccinic acid	4.92|236.09	UP	1.84	8.3 × 10^−10^
	N-Acetyl-L-phenylalanine	4.60|208.13	UP	0.51	5.9 × 10^−6^
	Diisodityrosine	5.07|716.85	UP	2.06	3.1 × 10^−7^
Coumarans	Carbosulfan	4.84|379.16	UP	4.00	2.5 × 10^−9^
Fatty Acyls	15-Palmitoylsolamin	4.33|801.42	UP	2.63	2.7 × 10^−4^
	Stearoyllactic acid	9.41|355.29	DOWN	0.59	3.5 × 10^−7^
	4,7,10,13-Hexadecatetraenoate	10.72|251.20	UP	0.60	4.1 × 10^−5^
Flavonoids	Eriodictin	5.70|433.21	UP	4.81	5.2 × 10^−11^
	7-Chloro-3,4′,5,6,8-pentamethoxyflavone	5.24|405.18	UP	4.58	5.3 × 10^−10^
Indoles & derivatives	Bismurrayafoline E	4.59|723.37	UP	1.99	5.4 × 10^−7^
	Indolepyruvate	4.84|204.10	UP	1.62	8.5 × 10^−4^
Organooxygen compounds	Salicyluric beta-D-glucuronide	3.90|370.08	UP	9.00	1.1 × 10^−15^
	Aldehydo-N-acetyl-D-glucosamine	5.38|219.95	UP	5.37	3.8 × 10^−10^
	N-Acetylgalactosamine 4-sulphate	4.99|302.20	UP	0.64	2.9 × 10^−4^
	alpha-Furyl methyl diketone	5.38|137.02	UP	7.18	3.2 × 10^−10^
Oxazinanes	Molsidomine	8.02|241.18	DOWN	0.53	3.9 × 10^−10^
Prenol lipids	15-cis-Phytoene	5.34|543.28	UP	2.18	6.1 × 10^−10^
	′-6′-O-(4-Geranyloxy-2-hydroxycinnamoyl)-marmin	5.08|631.35	UP	1.54	9.4 × 10^−5^
Pyrimidine nucleoside’	2′,2′-Difluorodeoxyuridine	4.66|264.01	UP	5.39	6.0 × 10^−13^
Pyrroles	O-Hydroxyatorvastatin	7.45|573.24	UP	2.98	1.6 × 10^−12^
Steroids & steroid derivatives	Etiocholanolone	10.39|241.18	UP	0.57	1.6 × 10^−5^
Tetrapyrroles & derivatives	L-Urobilin	5.73|593.34	UP	3.39	5.0 × 10^−4^

^a^ “UP” denotes presence of higher amounts of the analyte (i.e., up-regulated) in AMI patients as compared to healthy volunteers, while “DOWN” denotes the presence of lower amounts of the analyte (i.e., down-regulated) in AMI patients. ^b^ Fold-change was calculated by dividing the mean peak area of analyte in AMI patients over the mean peak area of analyte in healthy volunteers. A fold-change > 1 also implies up-regulation in AMI patients, while a fold change < 1 implies down-regulation. ^c^ *p*-value was FDR-adjusted.

**Table 3:** List of annotated significantly altered lipidomic features.

Class	Compound Name	RT|*m*/*z*	Regulation (Up/Down) ^a^	Fold-Change ^b^	*p*-Value ^c^
Carboxylic acids & derivatives	Pentasine	11.02|654.6	UP	1.51	4.7 × 10^−6^
Cholesteryl esters	CE(18:2(9Z,12Z))	14.7|666.62	DOWN	0.53	5.6 × 10^−6^
Diacylglycerols	DG(18:0/18:1(9Z)/0: 0)	15.39|605.55	UP	1.69	1.8 × 10^−6^
	DG(20:0/20:5(5Z,8Z,11Z,14Z,17Z)/0:0)	14.68|671.57	DOWN	0.39	9.9 × 10^−7^
	DG(24:1(15Z)/14:1(9Z)/0:0)	15.34|631.57	UP	1.89	1.4 × 10^−4^
Fatty acids & acylcarnitines	Decanoylcarnitine	1.12|316.25	DOWN	0.58	9.4 × 10^−7^
	Tetradecanal	1.54|230.25	DOWN	0.45	6.5 × 10^−7^
	Docosatrienoic acid	14.69|299.27	DOWN	0.36	4.1 × 10^−6^
	Oleic acid	2.51|302.3	DOWN	0.32	9.5 × 10^−10^
	Palmitic acid	10.64|615.5	UP	1.66	2.8 × 10^−6^
	9-Decenoylcarnitine	1|314.23	DOWN	0.61	3.0 × 10^−6^
	Dodecanoylcarnitine	1.68|344.28	DOWN	0.64	8.2 × 10^−6^
	(5Z,8Z)- Tetradecadienoylcarnitine	1.58|368.28	DOWN	0.58	3.9 × 10^−7^
	O-Linoleoylcarnitine	4.06|424.34	DOWN	0.60	6.7 × 10^−9^
Fatty acyls	Cohibin D	10.99|577.52	UP	1.51	1.9 × 10^−5^
Flavonoids	Apigenin 4′-[p-coumaroyl-(->2)-glucuronyl-(1->2)-glucuronide] 7-glucuronide	14.66|944.87	UP	1.53	1.2 × 10^−6^
	Malvidin 3-(6″-p-coumarylglucoside)	11.48|640.59	UP	1.57	2.5 × 10^−5^
Glycerolipids	MG(18:1(11Z)/0:0/0: 0)	11.02|339.29	UP	1.66	6.5 × 10^−5^
	MG(20:4(5Z,8Z,11Z, 14Z)/0:0/0:0)	2.44|396.31	DOWN	0.53	8.9 × 10^−7^
Glycero-phospholipids	PE(P-18:1(9Z)/18:2(9Z,12Z))	9.88|746.51	DOWN	0.64	3.8 × 10^−8^
	PE(P-18:1(9Z)/16:1(9Z))	10.16|698.51	DOWN	0.55	1.3 × 10^−9^
	PS(22:6(4Z,7Z,10Z,1 3Z,16Z,19Z)/20:3(8Z,11Z,14Z))	10.27|902.51	UP	1.54	1.7 × 10^−9^
	PS(22:6(4Z,7Z,10Z,13Z,16Z,19Z)/20:3(8Z,11Z,14Z))	10.27|852.5	UP	1.80	3.5 × 10^−13^
	PE(18:0/16:0)	10.49|700.52	DOWN	0.49	2.1 × 10^−5^
	PE(18:1(9Z)/18:0)	10.57|726.54	DOWN	0.61	3.1 × 10^−10^
	PC(22:0/15:0)	9.49|804.55	DOWN	0.62	4.4 × 10^−9^
	PC(22:0/18:0)	9.19|846.6	DOWN	0.57	5.2 × 10^−5^
	PE(20:4(8Z,11Z,14Z, 17Z)/18:0)	10.16|750.54	DOWN	0.55	1.0 × 10^−4^
	PE(16:0/18:1(9Z))	10.36|718.54	UP	1.97	5.9 × 10^−8^
	PC(18:4(6Z,9Z,12Z,15Z)/15:0)	11.66|740.53	DOWN	0.47	1.1 × 10^−5^
	PC(18:2(9Z,12Z)/14:0)	9.35|730.54	DOWN	0.65	5.9 × 10^−7^
	PC(20:4(5Z,8Z,11Z,14Z)s/16:0)	9.49|782.57	DOWN	0.65	4.6 × 10^−9^
Organic trisulfides	Pollinastanol	7.64|401.34	DOWN	0.42	1.0 × 10^−3^
Phenols	Betaxolol	1.46|308.12	DOWN	0.35	4.5 × 10^−2^
Prenol lipids	beta-Vatirenene	14.69|203.18	DOWN	0.53	1.8 × 10^−4^
	Monomenthyl succinate	14.7|257.23	DOWN	0.60	8.2 × 10^−5^
	Ubiquinone-4	3.79|472.34	DOWN	0.49	8.7 × 10^−6^
Steroids & steroid derivatives	Cholesterol	14.69|369.35	DOWN	0.60	8.2 × 10^−6^
	8-Dehydrocholesterol	14.1|367.34	DOWN	0.61	1.1 × 10^−3^
Triacylglycerols	TG(16:1(9Z)/18:0/20:1(11Z))	15.37|904.83	UP	1.58	8.0 × 10^−9^
	TG(17:0/18:1(9Z)/18:1(9Z))	15.07|890.82	UP	1.65	2.0 × 10^−5^
	TG(18:0/18:0/18:1(9 Z))	16.03|906.85	UP	1.64	9.2 × 10^−5^
	TG(18:0/18:0/20:4(5 Z,8Z,11Z,14Z))	16.02|911.81	UP	1.54	3.2 × 10^−5^
	TG(15:0/22:2(13Z,16 Z)/20:0)	15.33|946.88	UP	1.58	6.7 × 10^−9^
	TG(18:1(9Z)/18:1(9Z)/20:1(11Z))	15.33|930.85	UP	1.63	5.8 × 10^−8^
	TG(18:0/18:1(9Z)/20:0)	16.63|934.88	UP	1.76	2.7 × 10^−4^

^a^ “UP” denotes presence of higher amounts of the analyte (i.e., up-regulated) in AMI patients as compared to healthy volunteers, vice versa. ^b^ Fold-change was calculated by dividing the mean peak area of analyte in AMI patients over the mean peak area of analyte in healthy volunteers. A fold-change > 1 also implies up-regulation in AMI patients, while a fold change < 1 implies down-regulation. ^c^ *p*-value was FDR-adjusted.

**Table 4:** Performance of random forest models built using glycomics, metallomics, metabolomics, lipidomics, and multi-omics features in classifying between AMI and healthy.

Classifier	No. of Features Used	AUC_ROC_	CI
Glycomics	37	0.786	0.688–0.883
Metallomics	30	0.851	0.782–0.904
Metabolomics	27	0.836	0.744–0.930
Lipidomics	48	0.822	0.724–0.905
Multi-omics	Top 100 out of 142	0.953	0.911–0.987

## Data Availability

The data presented in this study are available in our previous glycomics and metallomics works [10,11] and in the supplementary material for the metabolomics/lipidomics data.

## Supplementary Materials

The following supporting information can be downloaded at: https://www.mdpi.com/article/10.3390/metabo12111080/s1, Supplementary Methods; Table S1: List of significantly altered metabolite features based on PLS-DA’s VIP score, Mann-Whitney test, and fold-change criteria; Table S2: List of significantly altered lipidomic features based on PLS-DA’s VIP score, Mann Whitney test, and fold-change criteria; Figure S1: Relevance network plot of highly correlated (*r* > 0.5; *p*-value < 0.05) significant features across all four omics layers (glycomics + metallomics + metabolomics + lipidomics); Figure S2: Tri-omics (glycomics + metallomics + lipidomics) relevance network plot of highly significant features correlat-ed (*r* > 0.5; *p*-value < 0.05); Figure S3: Tri-omics (glycomics + metallomics + metabolomics) relevance network plot of highly significant features cor-related (*r* > 0.5; *p*-value < 0.05); Figure S4: Bi-omics (glycomics + metallomics) relevance network plot of highly significant features correlated (*r* > 0.5; *p*-value < 0.05); Figure S5: Bi-omics (glycomics + lipidomics) relevance network plot of highly significant features correlated (*r* > 0.5; *p*-value < 0.05); Figure S6: Bi-omics (glycomics + metabolomics) relevance network plot of highly significant features correlated (*r* > 0.5; *p*-value < 0.05); Figure S7: Bi-omics (metallomics + lipidomics) relevance network plot of highly significant features correlated (*r* > 0.5; *p*-value < 0.05); Figure S8: Bi-omics (metallomics + metabolomics) relevance network plot of highly significant features correlated (*r* > 0.5; *p*-value < 0.05); Figure S9: Bi-omics (metabolomics + lipidomics) relevance network plot of highly significant features correlated (*r* > 0.5; *p*-value < 0.05).

## References

[1] Aydin S., Ugur K., Aydin S., Sahin I., Yardim M. (2019). Biomarkers in acute myocardial infarction: Current perspectives. Vasc. Health Risk Manag..

[2] Barron H.V., Harr S.D., Radford M., Wang Y., Krumholz H.M. (2001). The association between white blood cell count and acute myocardial infarction mortality in patients ≥65 years of age: Findings from the cooperative cardiovascular project. J. Am. Coll. Cardiol..

[3] Engstrom G., Melander O., Hedblad B. (2009). Leukocyte count and incidence of hospitalizations due to heart failure. Circ. Heart Fail..

[4] Lim S.Y., Selvaraji S., Lau H., Li S.F.Y. (2021). Application of omics beyond the central dogma in coronary heart disease research: A bibliometric study and literature review. Comput. Biol. Med..

[5] Sheikh M.O., Tayyari F., Zhang S., Judge M.T., Weatherly D.B., Ponce F.V., Wells L., Edison A.S. (2019). Correlations Between LC-MS/MS-Detected Glycomics and NMR-Detected Metabolomics in Caenorhabditis elegans Development. Front. Mol. Biosci..

[6] Zhou L., Xu J.-D., Zhou S.-S., Mao Q., Kong M., Shen H., Li X.Y., Duan S.M., Xu J., Li S.L. (2016). Integrating targeted glycomics and untargeted metabolomics to investigate the processing chemistry of herbal medicines, a case study on Rehmanniae Radix. J. Chromatogr. A.

[7] Igl W., Polašek O., Gornik O., Knežević A., Pučić M., Novokmet M., Huffman J., Gnewuch C., Liebisch G., Rudd P.M. (2011). Glycomics meets lipidomics—Associations of N-glycans with classical lipids, glycerophospholipids, and sphingolipids in three European populations. Mol. BioSyst..

[8] Wang M., Yu G., Ressom H.W. (2016). Integrative Analysis of Proteomic, Glycomic, and Metabolomic Data for Biomarker Discovery. IEEE J. Biomed. Health Inform..

[9] Lucio M., Willkommen D., Schroeter M., Sigaroudi A., Schmitt-Kopplin P., Michalke B. (2019). Integrative Metabolomic and Metallomic Analysis in a Case-Control Cohort with Parkinson’s Disease. Front. Aging Neurosci..

[10] Lim S.Y., Hendra C., Yeo X.H., Tan X.Y., Ng B.H., Laserna A.K.C., Tan S.H., Chan M.Y., Khan S.H., Chen S.M. (2022). N-glycan profiles of acute myocardial infarction patients reveal potential biomarkers for diagnosis, severity assessment and treatment monitoring. Glycobiology.

[11] Lim S.Y., Dayal H., Seah S.J., Tan R.P.W., Low Z.E., Laserna A.K., Tan S.H., Chan M.Y., Li S.F.Y. (2022). Plasma metallomics reveals potential biomarkers and insights into the ambivalent associations of elements with acute myocardial infarction. medRxiv.

[12] Matyash V., Liebisch G., Kurzchalia T.V., Shevchenko A., Schwudke D. (2008). Lipid extraction by methyl-tert-butyl ether for high-throughput lipidomics. J. Lipid Res..

[13] Patterson R.E., Ducrocq A.J., McDougall D.J., Garrett T.J., Yost R.A. (2015). Comparison of blood plasma sample preparation methods for combined LC–MS lipidomics and metabolomics. J. Chromatogr. B.

[14] Sostare J., Di Guida R., Kirwan J., Chalal K., Palmer E., Dunn W.B., Viant M.R. (2018). Comparison of modified Matyash method to conventional solvent systems for polar metabolite and lipid extractions. Anal. Chim. Acta.

[15] Dihazi H., Asif A.R., Beißbarth T., Bohrer R., Feussner K., Feussner I., Jahn O., Lenz C., Majcherczyk A., Schmidt B. (2018). Integrative omics—From data to biology. Expert Rev. Proteom..

[16] Ebrahim A., Brunk E., Tan J., O’Brien E.J., Kim D., Szubin R., Lerman J.A., Lechner A., Sastry A., Bordbar A. (2016). Multi-omic data integration enables discovery of hidden biological regularities. Nat. Commun..

[17] Yan J., Risacher S.L., Shen L., Saykin A.J. (2018). Network approaches to systems biology analysis of complex disease: Integrative methods for multi-omics data. Brief. Bioinform..

[18] Yugi K., Kubota H., Hatano A., Kuroda S. (2016). Trans-Omics: How to Reconstruct Biochemical Networks Across Multiple ‘Omic’ Layers. Trends Biotechnol..

[19] Konjevod M., Tudor L., Strac D.S., Erjavec G.N., Barbas C., Zarkovic N., Nikolac Perkovic M., Uzun S., Kozumplik O., Lauc G. (2019). Metabolomic and glycomic findings in posttraumatic stress disorder. Prog. Neuropsychopharmacol. Biol. Psychiatry.

[20] Kessner D., Chambers M., Burke R., Agus D., Mallick P. (2008). ProteoWizard: Open source software for rapid proteomics tools development. Bioinformatics.

[21] Salek R.M., Steinbeck C., Viant M.R., Goodacre R., Dunn W.B. (2013). The role of reporting standards for metabolite annotation and identification in metabolomic studies. GigaScience.

[22] Dunn W.B., Broadhurst D., Begley P., Zelena E., Francis-McIntyre S., Anderson N., Brown M., Knowles J.D., Halsall A., Haselden J.N. (2011). Procedures for large-scale metabolic profiling of serum and plasma using gas chromatography and liquid chromatography coupled to mass spectrometry. Nat. Protoc..

[23] Robin X., Turck N., Hainard A., Tiberti N., Lisacek F., Sanchez J.-C., Müller M. (2011). pROC: An open-source package for R and S+ to analyze and compare ROC curves. BMC Bioinform..

[24] Rohart F., Gautier B., Singh A., Lê Cao K.-A. (2017). mixOmics: An R package for ‘omics feature selection and multiple data integration. PLoS Comput. Biol..

[25] Naz S., Vallejo M., García A., Barbas C. (2014). Method validation strategies involved in non-targeted metabolomics. J. Chromatogr. A.

[26] Chin W.W. (1998). The partial least squares approach for structural equation modeling. Modern Methods for Business Research.

[27] Meikle P.J., Wong G., Tsorotes D., Barlow C.K., Weir J.M., Christopher M.J., Macintosh G.L., Goudey B., Stern L., Kowalczyk A. (2011). Plasma Lipidomic Analysis of Stable and Unstable Coronary Artery Disease. Arterioscler. Thromb. Vasc. Biol..

[28] Shah S.H., Sun J.-L., Stevens R.D., Bain J.R., Muehlbauer M.J., Pieper K.S., Haynes C., Hauser E.R., Kraus W.E., Granger C.B. (2012). Baseline metabolomic profiles predict cardiovascular events in patients at risk for coronary artery disease. Am. Heart J..

[29] Vaarhorst A.A., Verhoeven A., Weller C.M., Böhringer S., Göraler S., Meissner A., Deelder A.M., Henneman P., Gorgels A.P.M., Van Den Brandt P.A. (2014). A metabolomic profile is associated with the risk of incident coronary heart disease. Am. Heart J..

[30] Basak T., Varshney S., Hamid Z., Ghosh S., Seth S., Sengupta S. (2015). Identification of metabolic markers in coronary artery disease using an untargeted LC-MS based metabolomic approach. J. Proteom..

[31] Fan Y., Li Y., Chen Y., Zhao Y.-J., Liu L.-W., Li J., Wang S.-L., Alolga R.N., Yin Y., Wang X.-M. (2016). Comprehensive Metabolomic Characterization of Coronary Artery Diseases. J. Am. Coll. Cardiol..

[32] Li R., Li F., Feng Q., Liu Z., Jie Z., Wen B., Xu X., Zhong S., Li G., He K. (2016). An LC-MS based untargeted metabolomics study identified novel biomarkers for coronary heart disease. Mol. BioSyst..

[33] Xu X., Gao B., Guan Q., Zhang D., Ye X., Zhou L., Tong G., Li H., Zhang L., Tian J. (2016). Metabolomic profile for the early detection of coronary artery disease by using UPLC-QTOF/MS. J. Pharm. Biomed. Anal..

[34] Zhang X.-Z., Zheng S.-X., Hou Y.-M. (2017). A Non-Targeted Liquid Chromatographic-Mass Spectrometric Metabolomics Approach for Association with Coronary Artery Disease: An Identification of Biomarkers for Depiction of Underlying Biological Mechanisms. Med. Sci. Monit..

[35] Feng L., Yang J., Liu W., Wang Q., Wang H., Shi L., Fu L., Xu Q., Wang B., Li T. (2018). Lipid Biomarkers in Acute Myocardial Infarction Before and After Percutaneous Coronary Intervention by Lipidomics Analysis. Med. Sci. Monit..

[36] Huang L., Zhang L., Li T., Liu Y.-W., Wang Y., Liu B.-J. (2018). Human Plasma Metabolomics Implicates Modified 9-cis-Retinoic Acid in the Phenotype of Left Main Artery Lesions in Acute ST-Segment Elevated Myocardial Infarction. Sci. Rep..

[37] Kohlhauer M., Dawkins S., Costa A.S.H., Lee R., Young T., Pell V.R., Choudhury R.P., Banning A.P., Kharbanda R.K., Saeb-Parsy K. (2018). Metabolomic Profiling in Acute ST-Segment–Elevation Myocardial Infarction Identifies Succinate as an Early Marker of Human Ischemia–Reperfusion Injury. J. Am. Heart Assoc..

[38] Rämö J., Ripatti P., Tabassum R., Söderlund S., Matikainen N., Gerl M., Klose C., Surma M.A., Stitziel N.O., Havulinna A.S. (2019). Coronary Artery Disease Risk and Lipidomic Profiles Are Similar in Hyperlipidemias with Family History and Population-Ascertained Hyperlipidemias. J. Am. Heart Assoc..

[39] Barchuk M., Dutour A., Ancel P., Svilar L., Miksztowicz V., Lopez G., Rubio M., Schreier L., Nogueira J.P., Valéro R. (2020). Untargeted Lipidomics Reveals a Specific Enrichment in Plasmalogens in Epicardial Adipose Tissue and a Specific Signature in Coronary Artery Disease. Arterioscler. Thromb. Vasc. Biol..

[40] Gundogdu G., Senol O., Miloglu F.D., Koza Y., Gundogdu F., Hacımüftüoğlu A., Abd El-Aty A.M. (2020). Serum metabolite profiling of ST-segment elevation myocardial infarction using liquid chromatography quadrupole time-of-flight mass spectrometry. Biomed. Chromatogr..

[41] Mehta A., Liu C., Nayak A., Tahhan A.S., Ko Y.-A., Dhindsa D.S., Kim J.H., Hayek S.S., Sperling L.S., Mehta P.K. (2020). Untargeted high-resolution plasma metabolomic profiling predicts outcomes in patients with coronary artery disease. PLoS ONE.

[42] Deidda M., Noto A., Dessalvi C.C., Andreini D., Andreotti F., Ferrannini E., Latini R., Maggioni A.P., Magnoni M., Maseri A. (2021). Metabolomic correlates of coronary atherosclerosis, cardiovascular risk, both or neither. Results of the 2 × 2 phenotypic CAPIRE study. Int. J. Cardiol..

[43] Ottosson F., Khoonsari P.E., Gerl M.J., Simons K., Melander O., Fernandez C. (2021). A plasma lipid signature predicts incident coronary artery disease. Int. J. Cardiol..

[44] Huang M., Jiao J., Zhuang P., Chen X., Wang J., Zhang Y. (2018). Serum polyfluoroalkyl chemicals are associated with risk of cardiovascular diseases in national US population. Environ. Int..

[45] Zago A.M., Faria N.M.X., Fávero J.L., Meucci R.D., Woskie S., Fassa A.G. (2020). Pesticide exposure and risk of cardiovascular disease: A systematic review. Glob. Public Health.

[46] Fahy E., Subramaniam S., Brown H.A., Glass C.K., Merrill A.H., Murphy R.C., Raetz C.R.H., Russell D.W., Seyama Y., Shaw W. (2005). A comprehensive classification system for lipids. J. Lipid Res..

[47] Chen H., Wang Z., Qin M., Zhang B., Lin L., Ma Q., Liu C., Chen X., Li H., Lai W. (2021). Comprehensive Metabolomics Identified the Prominent Role of Glycerophospholipid Metabolism in Coronary Artery Disease Progression. Front. Mol. Biosci..

[48] Ford D.A. (2010). Lipid oxidation by hypochlorous acid: Chlorinated lipids in atherosclerosis and myocardial ischemia. Clin. Lipidol..

[49] Leßig J., Fuchs B. (2009). Plasmalogens in Biological Systems: Their Role in Oxidative Processes in Biological Membranes, their Contribution to Pathological Processes and Aging and Plasmalogen Analysis. Curr. Med. Chem..

[50] Zhu Q., Wu Y., Mai J., Guo G., Meng J., Fang X., Chen X., Liu C., Zhong S. (2022). Comprehensive Metabolic Profiling of Inflammation Indicated Key Roles of Glycerophospholipid and Arginine Metabolism in Coronary Artery Disease. Front. Immunol..

[51] Warfel J.D., Bermudez E.M., Mendoza T.M., Ghosh S., Zhang J., Elks C.M., Mynatt R., Vandanmagsar B. (2016). Mitochondrial fat oxidation is essential for lipid-induced inflammation in skeletal muscle in mice. Sci. Rep..

[52] Chiu H.-C., Kovacs A., Ford D.A., Hsu F.-F., Garcia R., Herrero P., Saffitz J.E., Schaffer J.E. (2001). A novel mouse model of lipotoxic cardiomyopathy. J. Clin. Investig..

[53] Schulze P.C., Drosatos K., Goldberg I.J. (2016). Lipid Use and Misuse by the Heart. Circ. Res..

[54] Singh A., Shannon C.P., Gautier B., Rohart F., Vacher M., Tebbutt S.J., Lê Cao K.-A. (2019). DIABLO: An integrative approach for identifying key molecular drivers from multi-omics assays. Bioinformatics.

[55] Zhang P., Woen S., Wang T., Liau B., Zhao S., Chen C., Yang Y., Song Z., Wormald M.R., Yu C. (2016). Challenges of glycosylation analysis and control: An integrated approach to producing optimal and consistent therapeutic drugs. Drug Discov. Today.

[56] Bournazos S., Ravetch J.V. (2017). Diversification of IgG effector functions. Int. Immunol..

[57] Bournazos S., Gupta A., Ravetch J.V. (2020). The role of IgG Fc receptors in antibody-dependent enhancement. Nat. Rev. Immunol..

[58] Kara S., Amon L., Lühr J.J., Nimmerjahn F., Dudziak D., Lux A. (2020). Impact of Plasma Membrane Domains on IgG Fc Receptor Function. Front. Immunol..

[59] Salvatore G., Bernoud-Hubac N., Bissay N., Debard C., Daira P., Meugnier E., Proamer F., Hanau D., Vidal H., Aricò M. (2015). Human monocyte-derived dendritic cells turn into foamy dendritic cells with IL-17A. J. Lipid Res..

[60] Gurbanov R., Bilgin M., Severcan F. (2016). Restoring effect of selenium on the molecular content, structure and fluidity of diabetic rat kidney brush border cell membrane. Biochim. Biophys. Acta Biomembr..

[61] Huang Z., Rose A.H., Hoffmann P.R. (2012). The Role of Selenium in Inflammation and Immunity: From Molecular Mechanisms to Therapeutic Opportunities. Antioxid. Redox Signal..

[62] Melcrová A., Pokorna S., Pullanchery S., Kohagen M., Jurkiewicz P., Hof M., Jungwirth P., Cremer P.S., Cwiklik L. (2016). The complex nature of calcium cation interactions with phospholipid bilayers. Sci. Rep..

[63] Hunter G.W., Negash S., Squier T.C. (1999). Phosphatidylethanolamine Modulates Ca-ATPase Function and Dynamics. Biochemistry.

[64] Ottolia M., Torres N., Bridge J.H.B., Philipson K.D., Goldhaber J.I. (2013). Na/Ca exchange and contraction of the heart. J. Mol. Cell. Cardiol..

[65] Eisner D., Caldwell J., Trafford A. (2013). Sarcoplasmic reticulum Ca-ATPase and heart failure 20 years later. Circ. Res..

[66] Kawase Y., Hajjar R.J. (2008). The cardiac sarcoplasmic/endoplasmic reticulum calcium ATPase: A potent target for cardiovascular diseases. Nat. Clin. Pract. Cardiovasc. Med..

[67] Poyton M.F., Sendecki A.M., Cong X., Cremer P.S. (2016). Cu2+ Binds to Phosphatidylethanolamine and Increases Oxidation in Lipid Membranes. J. Am. Chem. Soc..

[68] Gimenez M.S., Oliveros L.B., Gomez N.N. (2011). Nutritional Deficiencies and Phospholipid Metabolism. Int. J. Mol. Sci..

[69] Zavareh R.B., Lau K.S., Hurren R., Datti A., Ashline D.J., Gronda M., Cheung P., Simpson C.D., Liu W., Wasylishen A.R. (2008). Inhibition of the Sodium/Potassium ATPase Impairs N-Glycan Expression and Function. Cancer Res..

[70] Tokhtaeva E., Munson K., Sachs G., Vagin O. (2010). N-glycan-dependent quality control of the Na,K-ATPase beta(2) subunit. Biochemistry.

[71] Psychogios N., Hau D.D., Peng J., Guo A.C., Mandal R., Bouatra S., Sinelnikov I., Krishnamurthy R., Eisner R., Gautam B. (2011). The Human Serum Metabolome. PLoS ONE.

[72] Naser F.J., Mahieu N.G., Wang L., Spalding J.L., Johnson S.L., Patti G.J. (2018). Two complementary reversed-phase separations for comprehensive coverage of the semipolar and nonpolar metabolome. Anal. Bioanal. Chem..

[73] Ortmayr K., Causon T.J., Hann S., Koellensperger G. (2016). Increasing selectivity and coverage in LC-MS based metabolome analysis. TrAC Trends Anal. Chem..

[74] Lim S.Y., Ng B.H., Vermulapalli D., Lau H., Laserna A.K.C., Yang X., Tan S.H., Chan M.Y., Li S.F.Y. (2022). Simultaneous Polar Metabolite and N-Glycan Extraction Workflow for Joint-Omics Analysis: A Synergistic Approach for Novel Insights into Diseases. J. Proteome Res..

