# Supplementary material for: Multi-Omics Investigation into Acute Myocardial Infarction: An Integrative Method Revealing Interconnections amongst the Metabolome, Lipidome, Glycome, and Metallome
Source: Metabolites. 2022 Nov 8;12(11):1080. doi: 10.3390/metabo12111080 (PMC9693522; doi:10.3390/metabo12111080)
Supplement: Supplementary file 1 [file metabolites-12-01080-s001.zip › metabolites-1914446-supplementary.pdf]

SUPPLEMENTARY FILE

Multi-Omics Investigation into Acute Myocardial Infarction: An Integrative Method Revealing Interconnections amongst the Metabolome, Lipidome, Glycome, and Metallome

Si Ying Lim [1,2], Felicia Li Shea Lim [2], Inmaculada Criado-Navarro [2], Xin Hao Yeo [2], Hiranya Dayal [2], Sri Dhruti Vemulapalli [2], Song Jie Seah [2], Anna Karen Carrasco Laserna [2,3], Xiaoxun Yang [4], Sock Hwee Tan [4], Mark Y. Chan [4] and Sam Fong Yau Li [1,2,*]

[1] NUS Graduate School's Integrative Sciences & Engineering Programme (ISEP), National University of Singapore, Singapore 119077, Singapore
[2] Department of Chemistry, National University of Singapore, Singapore 117543, Singapore
[3] Central Instrumentation Facility (Laguna Campus), Office of the Vice President for Research and Innovation, De La Salle University, Manila 1004, Philippines
[4] Cardiovascular Research Institute, Yong Loo Lin School of Medicine, National University of Singapore, Singapore 117599, Singapore
* Correspondence: chmlifys@nus.edu.sg; Tel.: +65-6516-2681; Fax: +65-6779-1691

- Supplementary Methods
- Supplementary Tables S1-2
- Supplementary Figures S1-9

Supplementary Methods

1. Chemicals and reagents

For glycan extraction and analysis, Peptide:N-glycosidase F (PNGaseF), magnetic beads, their associated buffers (digestion buffer for PNGase F digestion and wash buffer for magnetic beads-based purification respectively), and size standards were purchased from Thermo Fisher Scientific as part of their GlycanAssure™ HyPerformance APTS kit with CE module (catalogue number: A38927). 8-aminopyrene-1,3,6-trisulfonic acid (APTS) and 2-picoline borane were purchased from Sigma-Aldrich. APTS Maltodextrin ladder used for the generation of glucose unit (GU) calibration curve was purchased from Agilent Technologies (Santa Clara, CA, USA). Exoglycosidases and glycan standards used for glycan identification were purchased from New England Biolabs (Ipswich, MA, USA) and Dextra Laboratories (Reading, UK) respectively.

For elemental extraction and analysis, the multi-element standard (IV-ICPMS-71A) was purchased from Inorganic Ventures (Christiansburg, VA, USA), and it contains the following elements in 3% nitric acid: Ag, Al, As, B, Ba, Be, Ca, Cd, Ce, Co, Cr, Cs, Cu, Dy, Er, Eu, Fe, Ga, Gd, Ho, K, La, Lu, Mg, Mn, Na, Nd, Ni, P, Pb, Pr, Rb, S, Se, Sm, Sr, Th, Ti, Tm, U, V, Yb, Zn. The inductively coupled plasma-mass spectrometry (ICP-MS) internal standard mix was purchased from Agilent Technologies (contains Bi, Ge, In, Li-6, Rh, Sc and Tb). The 60% nitric acid (ultrapure; any metals at sub ppt levels) used for all experiments was purchased from Kanto Chemical Co. (Tokyo, Japan).

2. Glycomics sample preparation and CE-LIF analysis

Extraction and preparation of N-glycans for CE-LIF were performed as described in our previous work with modification.[56] Briefly, N-glycans in 2 µL of blood plasma sample were released via the addition of 0.75 µL of PNGase F and 1.5 µL of digestion buffer and incubating at 55 °C in a heat block. Released glycans were labelled with a fluorescence tag by adding 10 µL of APTS (20 mM in 3.6 M citric acid) and 10 µL of 2-picolane borane (0.2M in DMSO) and incubating at 37 °C for 18 h in a heat block. Solid-phase extraction of the released glycans with magnetic beads was done by adding 50 µL of magnetic beads and

185 μL of acetonitrile and captured with the aid of a magnetic stand. Purification of captured glycans was done with three washes of 100 μL wash buffer. Purified glycans for CE-LIF analysis were then eluted with 25 μL of elution buffer.

N-glycan samples were analyzed using a CE-LIF (Applied Biosystems™ 3500xL Genetic Analyzer, Thermo Fisher Scientific). Briefly, a CE loading matrix was prepared by adding 2 μL GeneScan™ 600 LIZ™ dye size standard and 2 μL of Landmark Red in 200 μL CE loading buffer, and incubated at 60 °C for 5 min. Analysis was then done with 2 μL of the purified glycan sample and 8 μL of the CE loading matrix added to each well of a 96-well reaction plate.

Data acquisition was done using the Applied Biosystems™ GlycanAssure™ Glycan Acquisition Software. The peaks generated were processed with the accompanying Data Analysis Software. To account for run-to-run variation, peak areas used for further statistical analysis were normalised to the total area of the measured peaks.

3. Metallomics sample preparation and ICP-MS/MS analysis

To obtain elemental digestates from human blood plasma, acid digestion aided with heating via a hotplate was done. Specifically, 80 μL blood plasma was added into a 15 mL polypropylene centrifuge tube, followed by the addition of 80 μL ultrapure water and 80 μL ultrapure nitric acid. The centrifuge tubes were gently swirled to allow mixing. Immediately after, the samples were heated using a hotplate at 98 °C for 2 hours. To further process the samples for ICP-MS analysis, the digested samples were diluted to 8 mL with ultrapure water. The diluted samples were then filtered using 2.2 μm PTFE syringe filters into clean centrifuge tubes. Blank samples were prepared in the same way, but with the blood plasma component replaced with ultrapure water. The samples were then stored at 4 °C until ICP-MS analysis.

ICP-MS analysis was done using the 8900 ICP-MS/MS system (equipped with a triple quadruple mass spectrometer) from Agilent Technologies. To correct for matrix effects and non-spectral interferences, multiple elements from the internal standard mix were spiked online and elemental concentrations were normalised to the internal standards. To obtain optimal precision and accuracy, the internal standard analyte for each element was selected as close in mass number as possible to that of the analyte element, with ionisation energies considered as well. To obtain elemental concentrations of the samples, external standard calibration was done using the multi-element standard prepared in various concentrations (0, 0.1, 0.5, 1.0, 10.0, 100.0, 500.0, 1000.0 ppb). For optimal accuracy using a 6-point calibration curve, the lower-most points (0.1 and 0.5 ppb) were removed for external calibration curve generation for elements with higher concentrations, while the upper-most points (500 and 1000 ppb) were removed from external calibration curve generation for elements with concentrations lower than 10 ppb. It was noted that a few elements (Al, Ca, K, Mg, Na, P, S) were present in higher abundance where their concentrations in some or all the plasma samples exceed the external standard calibration range. To determine their concentrations, the samples were diluted (dilution factor = 50) and re-analysed by the ICP-MS.

Table S1. List of significantly altered metabolite features based on PLS-DA's VIP score, Mann-Whitney test, and fold-change criteria.

RT|*m/z*	VIP (PC1)	VIP (PC2)	Fold-change	*p*-value	Level of identification	Formula	Compound name	Class	HMDB code
6.83|472.29	2.32	1.97	0.39	6.0×10^{-5}	2	C27H21ClFN3O2	Lixivaptan	2-arylbenzofuran flavonoids	HMDB0254131
6.41|613.36	2.04	1.73	1.85	7.6×10^{-9}	2	C11F23CO2H	Perfluorododecanoic acid	Alkyl halides	HMDB0256318
10.18|413.33	2.66	2.26	0.64	2.1×10^{-6}	2	C19H25Cl2N3O3	1-Piperazinecarboxylic acid, 4-((3,4-dichlorophenyl)acetyl)-3-(1-pyrrolidinylmethyl)-, methyl ester, (3R)-	Benzene and substituted derivatives	HMDB0252928
4.57|283.08	2.21	1.87	1.86	1.3×10^{-5}	2	C13H16O7	D-Vacciniin	Benzene and substituted derivatives	HMDB0038492
4.92|236.09	2.08	0.18	1.84	8.3×10^{-10}	2	C7H11NO6S	S-Cysteinosuccinic acid	Carboxylic acids and derivatives	HMDB0029418
4.60|208.13	2.20	1.99	0.51	5.9×10^{-6}	2	C11H13NO3	N-Acetyl-L-phenylalanine	Carboxylic acids and derivatives	HMDB0000512
5.07|716.85	2.32	2.06	2.06	3.1×10^{-7}	2	C36H38N4O12	Diisodityrosine	Carboxylic acids and derivatives	HMDB0029798
4.84|379.16	3.18	2.81	4.00	2.5×10^{-9}	2	C20H32N2O3S	Carbosulfan	Coumarans	HMDB0249647
4.33|801.42	2.06	1.75	2.63	2.7×10^{-4}	2	C51H94O6	15-Palmitoylsolamin	Fatty Acyls	HMDB0036341
9.41|355.29	2.08	1.78	0.59	3.5×10^{-7}	2	C21H40O4	Stearoyllactic acid	Fatty Acyls	HMDB0033372
10.72|251.20	2.06	1.57	0.60	4.1×10^{-5}	2	C16H26O2	4,7,10,13-Hexadecatetraenoate	Fatty Acyls	HMDB0246620
5.70|433.21	3.58	3.15	4.81	5.2×10^{-11}	2	C21H22O10	Eriodictin	Flavonoids	HMDB0037480
5.24|405.18	3.14	2.72	4.58	5.3×10^{-10}	2	C20H19ClO7	7-Chloro-3,4',5,6,8-pentamethoxyflavone	Flavonoids	HMDB0033069
4.59|723.37	2.26	2.07	1.99	5.4×10^{-7}	2	C48H56N2O4	Bismurrayafoline E	Indoles and derivatives	HMDB0037741
4.84|204.10	1.69	1.42	1.62	8.5×10^{-4}	2	C11H9NO3	Indolepyruvate	Indoles and derivatives	HMDB0060484
5.71|591.32	1.88	1.52	2.00	2.3×10^{-3}	2	C32H48O10	Aplysiatoxin, 17-debromo-	Macrolides and analogues	HMDB0248517
3.90|370.08	4.69	4.15	9.00	1.1×10^{-15}	2	C15H17NO10	Salicyluric beta-D-glucuronide	Organooxygen compounds	HMDB0240252
5.38|219.95	3.76	3.32	5.37	3.8×10^{-10}	2	C8H15NO6	Aldehydo-N-acetyl-D-glucosamine	Organooxygen compounds	HMDB0062641
4.99|302.20	1.85	1.44	0.64	2.9×10^{-4}	2	C8H15NO9S	N-Acetylgalactosamine 4-sulphate*	Organooxygen compounds	HMDB0000781

5.38|137.02	4.19	3.64	7.18	3.2×10^{-10}	2	C7H6O3	alpha-Furyl methyl diketone	Organooxygen compounds	HMDB0032920
8.02|241.18	2.35	2.15	0.53	3.9×10^{-10}	2	C9H14N4O4	1-Ethoxy-N-(3-morpholin-4-yloxadiazol-3-ium-5-yl)methanimidate	Oxazinanes	HMDB0245703
5.34|543.28	2.25	2.01	2.18	6.1×10^{-10}	2	C40H64	15-cis-Phytoene	Prenol lipids	HMDB39093
5.08|631.35	1.46	1.11	1.54	9.4×10^{-5}	2	C38H46O8	(R)-6'-O-(4-Geranyloxy-2-hydroxycinnamoyl)-marmin	Prenol lipids	HMDB0039061
4.66|264.01	4.19	3.68	5.39	6.0×10^{-13}	2	C9H10F2N2O5	2',2'-Difluorodeoxyuridine	Pyrimidine nucleosides	HMDB0060727
7.45|573.24	4.32	3.84	2.98	1.6×10^{-12}	2	C33H35FN2O6	O-Hydroxyatorvastatin	Pyrroles	HMDB0061015
10.39|241.18	2.13	1.59	0.57	1.6×10^{-5}	2	C19H30O2	Etiocholanolone	Steroids and steroid derivatives	HMDB0000490
5.73|593.34	2.27	1.95	3.39	5.0×10^{-4}	2	C33H46N4O6	L-Urobilin	Tetrapyrroles and derivatives	HMDB0240259
4.62|202.11	2.02	1.75	1.74	1.9×10^{-9}	4				
5.16|319.01	2.38	2.04	1.86	2.0×10^{-5}	4				
7.18|213.15	2.07	1.81	0.55	3.8×10^{-7}	4				
7.64|239.16	2.48	2.22	0.51	2.4×10^{-9}	4				
5.51|609.25	2.21	1.90	2.05	1.4×10^{-7}	4				
6.27|537.33	2.26	1.98	2.59	5.5×10^{-7}	4				
7.00|653.19	3.32	2.88	5.16	5.0×10^{-9}	4				
7.55|215.16	3.16	2.92	0.45	1.3×10^{-9}	4				
4.66|150.06	3.90	3.40	4.22	0.0×0.0	4				
5.65|835.40	2.73	2.16	4.31	1.4×10^{-4}	4				
7.78|325.18	1.57	1.27	1.56	3.4×10^{-3}	4				
10.09|409.33	1.93	1.49	0.66	1.6×10^{-4}	4				
10.52|135.12	1.69	1.30	0.65	5.3×10^{-4}	4				

Table S2. List of significantly altered lipidomic features based on PLS-DA's VIP score, Mann Whitney test, and fold-change criteria.

**RT|*m/z***	**Regulation (up/down)**	**VIP (PC1)**	**VIP (PC2)**	**Fold-change**	***p*-value**	**Level of identification**	**Formula**	**Compound name**	**Class**	**HMDB code**
1.44|540.36	DOWN	2.7407	2.788	0.53	1.2×10^{-5}	2	C22H27ClFN7O4S	Encorafenib	Azoles	HMDB0304887
4.46|277.14	DOWN	5.4309	5.2609	0.32	3.5×10^{-17}	2	C16H22O4	Monoethylhexyl phthalic acid	Benzene and substirtued derivatives	HMDB0013248
15.3|972.9	UP	2.2544	1.9839	1.64	7.9×10^{-8}	2	C20H4Cl4I4O5	Rose bengal	Benzopyrans	HMDB0036613
11.02|654.6	UP	2.0782	1.9047	1.51	4.7×10^{-6}	2	C30H49N6O10	Pentasine	Carboxylic acids and derivatives	HMDB0029803
14.7|666.62	DOWN	2.3483	2.1523	0.53	5.6×10^{-6}	2	C45H76O2	CE(18:2(9Z,12Z))	Cholesteryl Esters	HMDB0000610
10.64|615.5	UP	2.3945	2.1185	1.66	2.8×10^{-6}	2	C37H68O5	palmitic acid	Diacylglycerols	HMDB0007131
15.39|605.55	UP	2.7135	2.4523	1.69	1.8×10^{-6}	2	C39H74O5	DG(18:0/18:1(9Z)/0: 0)	Diacylglycerols	HMDB0007160
1.12|316.25	DOWN	2.51	2.3011	0.58	9.4×10^{-7}	2	C17H33NO4	Decanoylcarnitine	Fatty Acids	HMDB0000651
1.54|230.25	DOWN	3.2182	3.1133	0.45	6.5×10^{-7}	2	C14H28O	Tetradecanal	Fatty Acids	HMDB0034283
14.69|299.27	DOWN	2.7342	2.4149	0.36	4.1×10^{-6}	2	C22H38O2	Docosatrienoic acid	Fatty Acids	HMDB0002823
2.51|302.3	DOWN	4.0751	3.8635	0.32	9.5×10^{-10}	2	C18H34O2	Oleic acid	Fatty Acids	HMDB0000207
1|314.23	DOWN	2.3252	2.1076	0.61	3.0×10^{-6}	2	C17H31NO4	9-Decenoylcarnitine	Fatty Acids	HMDB0013205
1.68|344.28	DOWN	2.1483	2.0289	0.64	8.2×10^{-6}	2	C19H37NO4	Dodecanoylcarnitine	Fatty Acids	HMDB0002250
1.58|368.28	DOWN	2.5025	2.312	0.58	3.9×10^{-7}	2	C21H37NO4	(5Z,8Z)- Tetradecadienoylcarn -itine	Fatty Acids	HMDB0240756
4.06|424.34	DOWN	2.4197	2.2101	0.60	6.7×10^{-9}	2	C25H45NO4	O-Linoleoylcarnitine	Fatty Acids	HMDB0240780
10.99|577.52	UP	2.0163	1.8505	1.51	1.9×10^{-5}	2	C37H68O4	Cohibin D*	Fatty Acyls	HMDB0035398
14.66|944.87	UP	2.0494	1.8162	1.53	1.2×10^{-6}	2	C42H40O25	Apigenin 4'-[p-coumaroyl-(->2)-glucuronyl-(1->2)-glucuronide] 7-glucuronide	Flavonoids	HMDB0038296
11.48|640.59	UP	2.2741	2.119	1.57	2.5×10^{-5}	2	C32H31O14	Malvidin 3-(6"-p-coumarylglucoside)	Flavonoids	HMDB0301903
14.68|671.57	DOWN	2.9795	2.7036	0.39	9.9×10^{-7}	2	C43H74O5	DG(20:0/20:5(5Z,8Z,11Z,14Z,17Z)/0:0)	Glycerolipids	HMDB0007375
11.02|339.29	UP	2.3923	2.254	1.66	6.5×10^{-5}	2	C21H40O4	MG(18:1(11Z)/0:0/0: 0)	Glycerolipids	HMDB0011566
2.44|396.31	DOWN	2.9146	2.6465	0.53	8.9×10^{-7}	2	C23H38O4	MG(20:4(5Z,8Z,11Z, 14Z)/0:0/0:0)	Glycerolipids	HMDB0011578

9.88|746.51	DOWN	2.2165	2.0069	0.64	3.8×10^{-8}	2	C41H76NO7P	PE(P-18:1(9Z)/18:2(9Z,12Z))	Glycerophospholipids	HMDB0011442
10.16|698.51	DOWN	2.1331	1.9399	0.55	1.3×10^{-9}	2	C39H74NO7P	PE(P-18:1(9Z)/16:1(9Z))	Glycerophospholipids	HMDB0011438
10.27|902.51	UP	2.3498	2.1532	1.54	1.7×10^{-9}	2	C48H76NO10P	PS(22:6(4Z,7Z,10Z,1 3Z,16Z,19Z)/20:3(8Z ,11Z,14Z))	Glycerophospholipids	HMDB0012448
10.27|852.5	UP	2.8868	2.6142	1.80	3.5×10^{-13}	2	C44H74NO10P	PS(22:6(4Z,7Z,10Z,13Z,16Z,19Z)/20:3(8Z,11Z,14Z))	Glycerophospholipids	HMDB0012442
10.49|700.52	DOWN	2.8415	2.8256	0.49	2.1×10^{-5}	2	C39H76NO7P	PE(18:0/16:0)	Glycerophospholipids	HMDB0008989
10.57|726.54	DOWN	2.5896	2.3485	0.61	3.1×10^{-10}	2	C41H80NO8P	PE(18:1(9Z)/18:0)	Glycerophospholipids	HMDB0009057
15.34|631.57	UP	2.3813	2.1827	1.89	1.4×10^{-4}	2	C41H76O5	DG(24:1(15Z)/14:1(9Z)/0:0)*	Glycerophospholipids	HMDB0007821
9.49|804.55	DOWN	2.3011	2.1476	0.62	4.4×10^{-9}	2	C45H90NO8P	PC(22:0/15:0)	Glycerophospholipids	HMDB0008527
9.19|846.6	DOWN	2.2381	2.0012	0.57	5.2×10^{-5}	2	C48H96NO8P	PC(22:0/18:0)	Glycerophospholipids	HMDB0008530
10.16|750.54	DOWN	2.2684	2.2338	0.55	1.0×10^{-4}	2	C43H78NO8P	PE(20:4(8Z,11Z,14Z, 17Z)/18:0)	Glycerophospholipids	HMDB0009420
10.36|718.54	UP	3.0927	2.7182	1.97	5.9×10^{-8}	2	C39H76NO8P	PE(16:0/18:1(9Z))	Glycerophospholipids	HMDB0008927
11.66|740.53	DOWN	2.5112	2.2883	0.47	1.1×10^{-5}	2	C39H76NO8P	PC(18:4(6Z,9Z,12Z,15Z)/15:0)*	Glycerophospholipids	HMDB0009250
9.35|730.54	DOWN	2.0865	2.062	0.65	5.9×10^{-7}	2	C40H76NO8P	PC(18:2(9Z,12Z)/14:0)*	Glycerophospholipids	HMDB0008130
11.66|740.53	DOWN	2.5112	2.2883	0.47	1.1×10^{-5}	2	C41H74NO8P	PC(18:4(6Z,9Z,12Z,15Z)/15:0)	Glycerophospholipids	HMDB0008231
9.49|782.57	DOWN	2.1568	2.0107	0.65	4.6×10^{-9}	2	C44H80NO8P	PC(20:4(5Z,8Z,11Z,14Z)/16:0)	Glycerophospholipids	HMDB0008429
7.64|401.34	DOWN	2.2936	2.6701	0.42	1.0×10^{-3}	2	C28H48O	Pollinastanol	Organic trisulfides	HMDB0034418
1.46|308.12	DOWN	1.9283	2.0061	0.35	4.5×10^{-2}	2	C18H29NO3	Betaxolol	Phenols	HMDB0014341
14.69|203.18	DOWN	2.3877	2.2515	0.53	1.8×10^{-4}	2	C15H22	beta-Vatirenene	Prenol Lipids	HMDB0059676
14.7|257.23	DOWN	2.2154	2.0102	0.60	8.2×10^{-5}	2	C14H24O4	Monomenthyl succinate	Prenol lipids	HMDB0036143
3.79|472.34	DOWN	2.9761	2.6682	0.49	8.7×10^{-6}	2	C29H42O4	Ubiquinone-4	Prenol Lipids	HMDB0006710
14.69|369.35	DOWN	2.063	1.8893	0.60	8.2×10^{-6}	2	C27H46O	Cholesterol	Steroids and Steroid derivatives	HMDB0000067
14.1|367.34	DOWN	2.0602	1.9187	0.61	1.1×10^{-3}	2	C27H44O	8-Dehydrocholesterol	Steroids and Steroid derivatives	HMDB0006842
15.37|904.83	UP	2.2741	2.0304	1.58	8.0×10^{-9}	2	C57H106O6	TG(16:1(9Z)/18:0/20 :1(11Z))	Triacylglycerols	HMDB0005424
15.07|890.82	UP	2.0449	1.9007	1.65	2.0×10^{-5}	2	C56H104O6	TG(17:0/18:1(9Z)/18 :1(9Z))	Triacylglycerols	HMDB0062701
16.03|906.85	UP	2.2827	2.1978	1.64	9.2×10^{-5}	2	C57H108O6	TG(18:0/18:0/18:1(9 Z))	Triacylglycerols	HMDB0005395

16.02|911.81	UP	2.125	2.0093	1.54	3.2×10^{-5}	2	C59H106O6	TG(18:0/18:0/20:4(5 Z,8Z,11Z,14Z))	Triacylglycerols	HMDB0005398
15.33|946.88	UP	2.286	2.0256	1.58	6.7×10^{-9}	2	C60H112O6	TG(15:0/22:2(13Z,16 Z)/20:0)	Triacylglycerols	HMDB0043543
15.33|930.85	UP	2.209	1.9492	1.63	5.8×10^{-8}	2	C59H108O6	TG(18:1(9Z)/18:1(9Z)/20:1(11Z))	Triacylglycerols	HMDB0005454
16.63|934.88	UP	2.2138	2.0893	1.76	2.7×10^{-4}	2	C59H112O6	TG(18:0/18:1(9Z)/20 :0)	Triacylglycerols	HMDB0005402
14.7|219.21	DOWN	2.0704	1.9939	0.60	8.5×10^{-4}	4				
14.7|233.23	DOWN	2.6706	2.4444	0.49	3.2×10^{-5}	4				
14.69|243.21	DOWN	2.437	2.2852	0.53	1.5×10^{-4}	4				
14.7|271.24	DOWN	2.7088	2.4347	0.51	3.2×10^{-5}	4				
11.02|595.53	UP	2.728	2.5127	1.76	1.2×10^{-5}	4				
9.1|802.53	DOWN	2.1664	2.0032	0.55	3.6×10^{-5}	4				
15.42|861.79	UP	2.0925	1.997	1.53	2.8×10^{-4}	4				
15.57|903.82	UP	2.2611	2.0262	1.63	8.9×10^{-6}	4				
15.68|923.8	UP	2.3777	2.1677	2.32	1.7×10^{-4}	4				

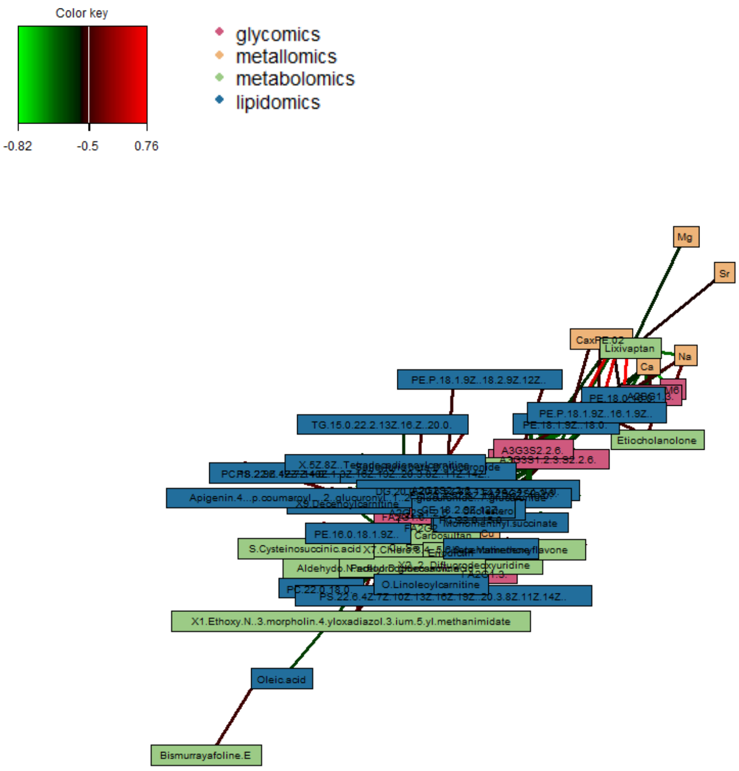

Figure S1. Relevance network plot of highly correlated ($r > 0.5$; p-value < 0.05) significant features across all four omics layers (glycomics + metallomics + metabolomics + lipidomics).

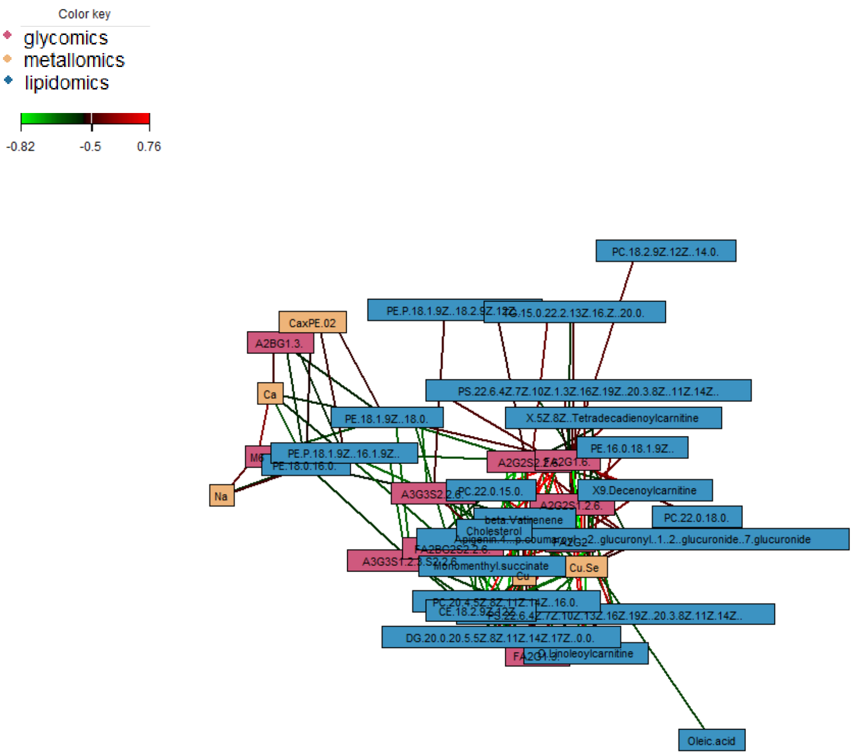

Figure S2. Tri-omics (glycomics + metallomics + lipidomics) relevance network plot of highly significant features correlated ($r > 0.5$; p-value < 0.05).

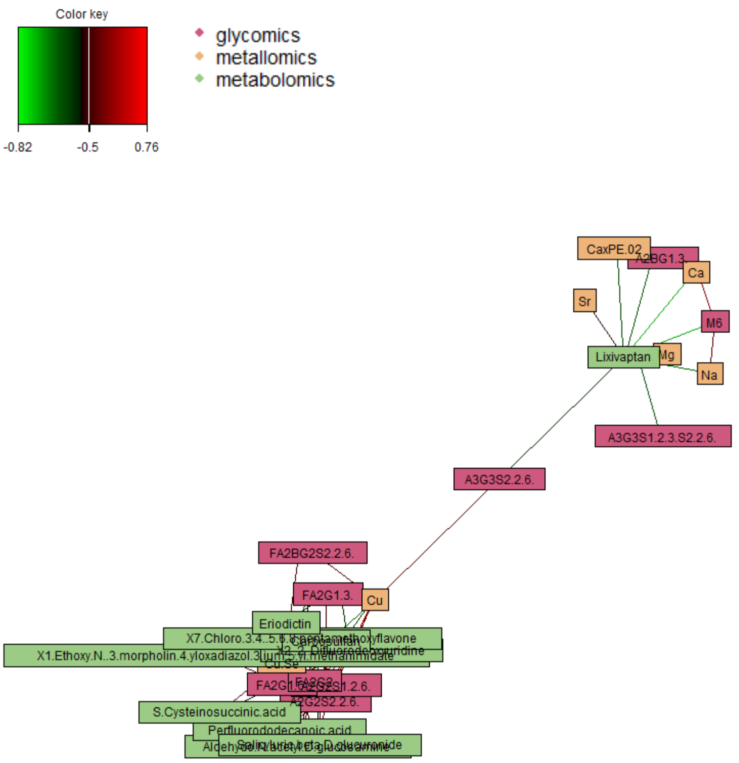

Figure S3. Tri-omics (glycomics + metallomics + metabolomics) relevance network plot of highly significant features correlated (*r* > 0.5; *p*-value < 0.05).

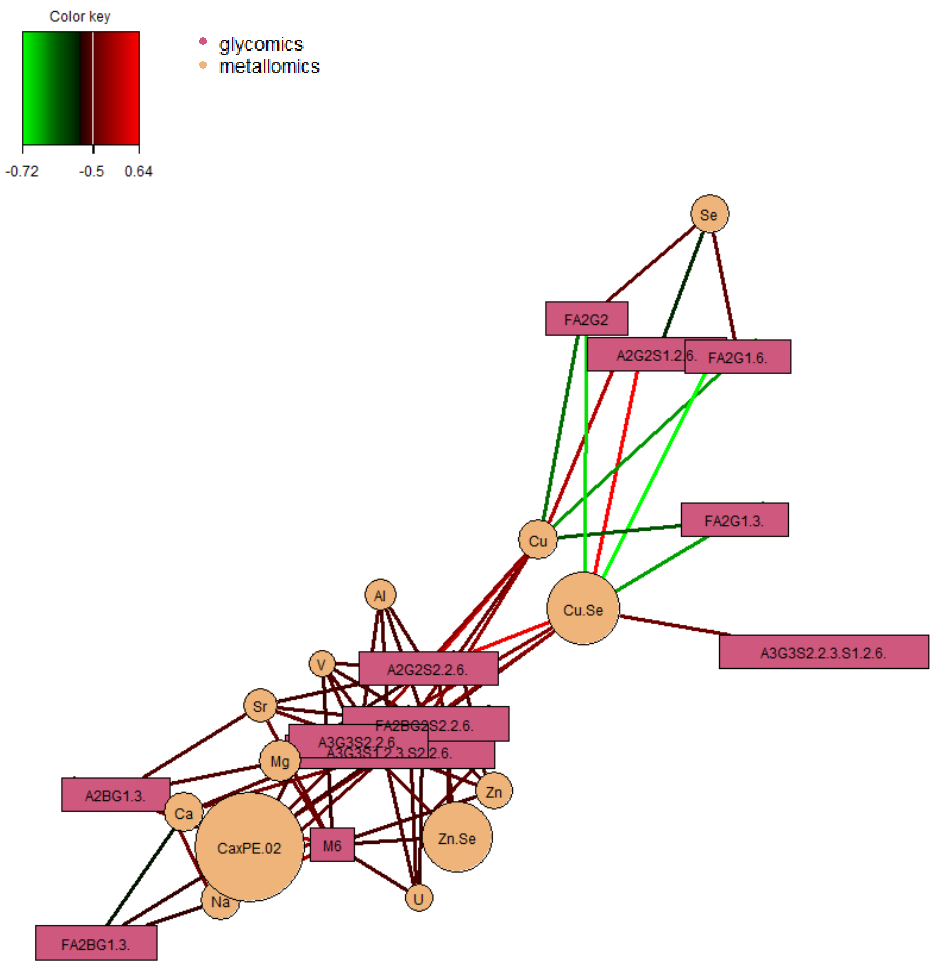

Figure S4. Bi-omics (glycomics + metallomics) relevance network plot of highly significant features correlated (r > 0.5; p-value < 0.05).

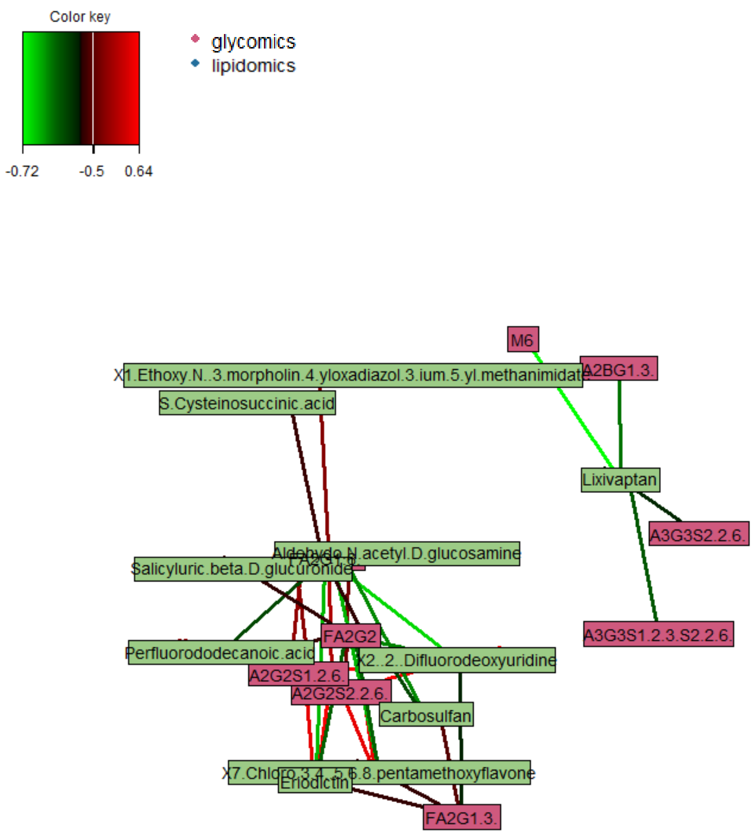

Figure S5. Bi-omics (glycomics + lipidomics) relevance network plot of highly significant features correlated ($r > 0.5$; p-value < 0.05).

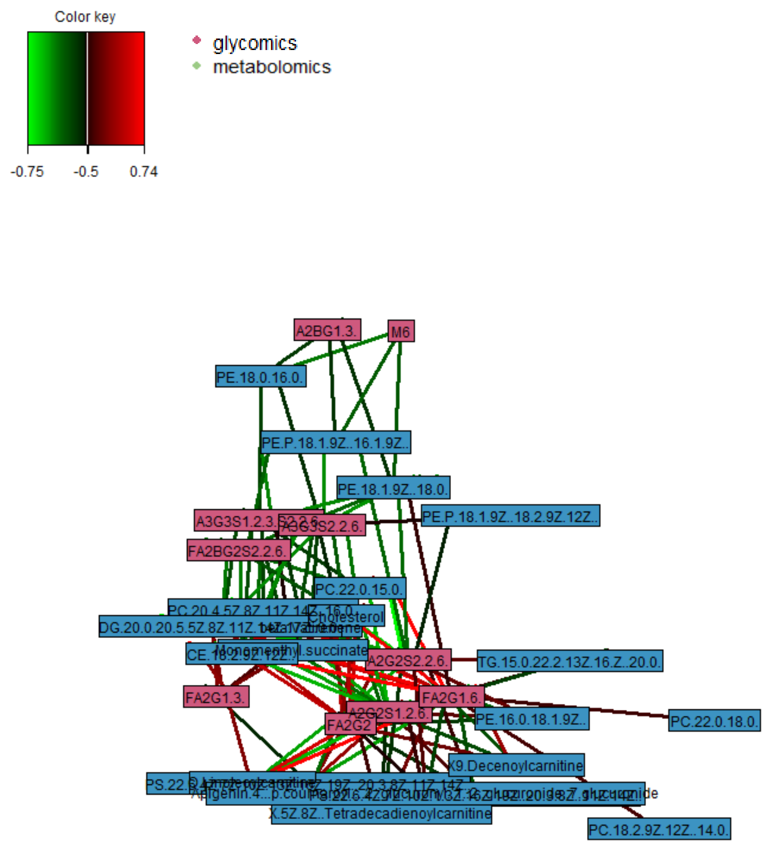

Figure S6. Bi-omics (glycomics + metabolomics) relevance network plot of highly significant features correlated (*r* > 0.5; *p*-value < 0.05).

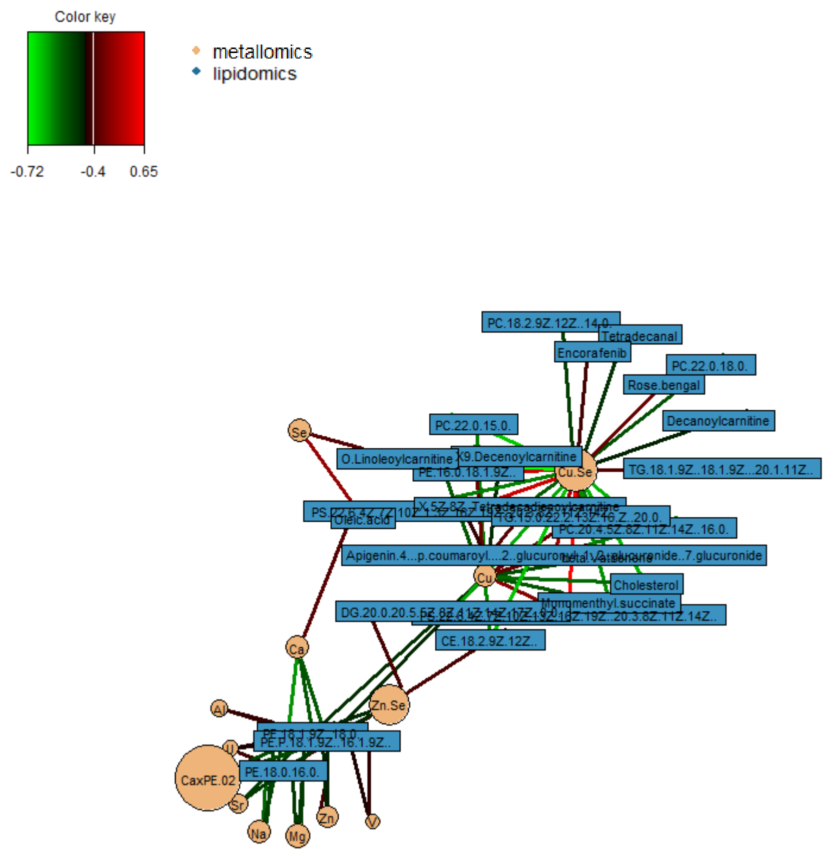

Figure S7. Bi-omics (metallomics + lipidomics) relevance network plot of highly significant features correlated ($r > 0.5$; p-value < 0.05).

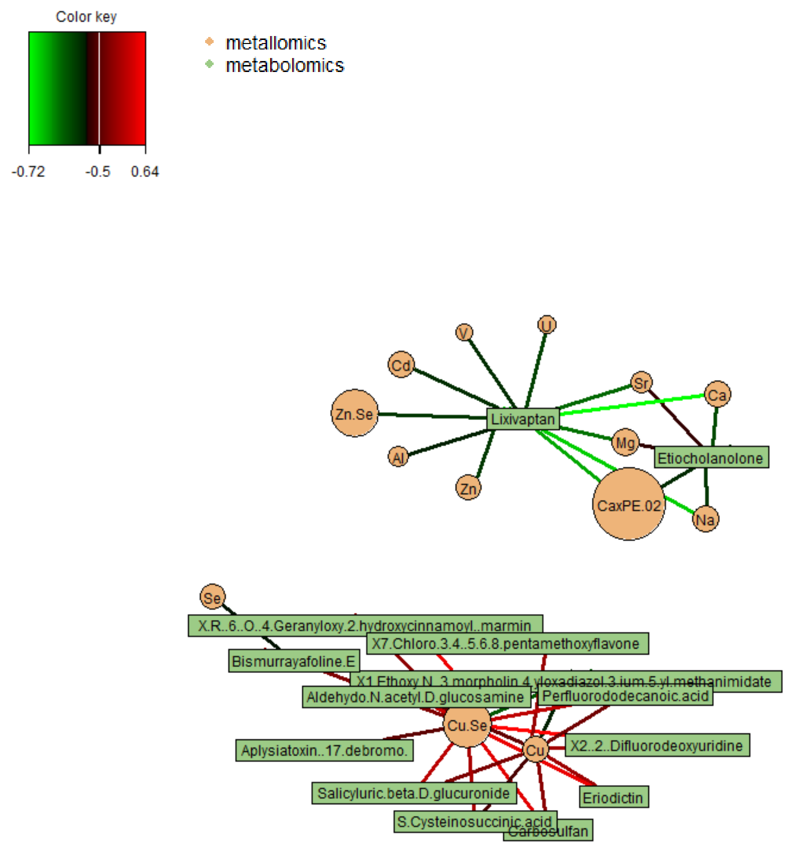

Figure S8. Bi-omics (metallomics + metabolomics) relevance network plot of highly significant features correlated ($r > 0.5$; p-value < 0.05).

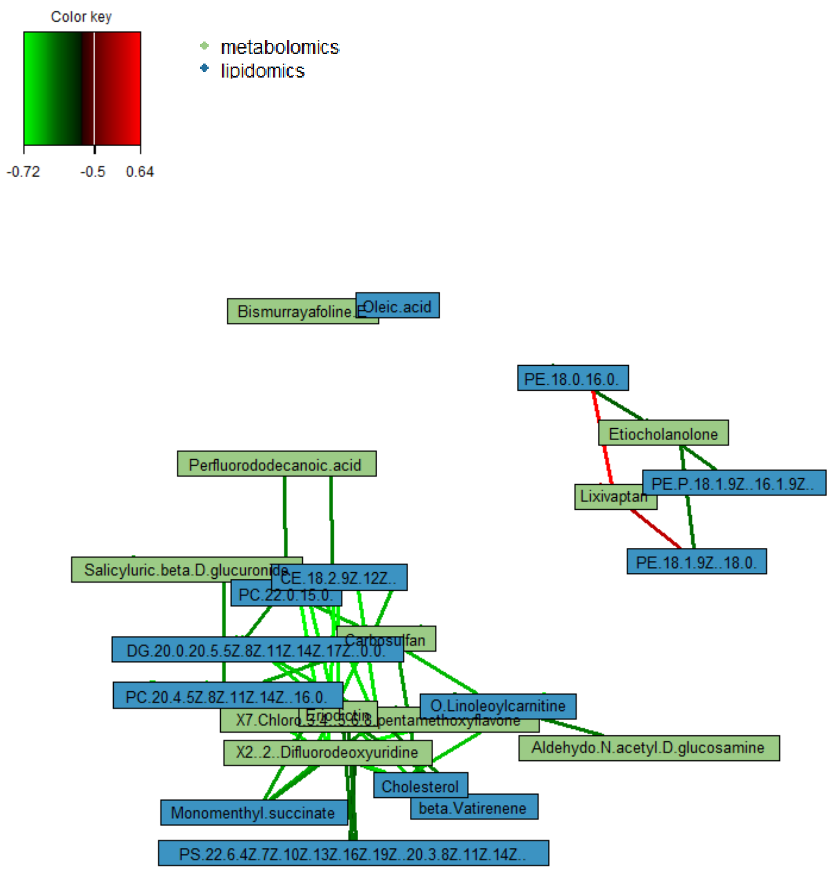

Figure S9. Bi-omics (metabolomics + lipidomics) relevance network plot of highly significant features correlated ($r > 0.5$; p-value < 0.05).
